# First-principles investigation of the lattice vibrations in the alkali feldspar solid solution

**DOI:** 10.1007/s00269-014-0715-8

**Published:** 2014-10-11

**Authors:** Artur Benisek, Edgar Dachs, Michael Grodzicki

**Affiliations:** Materialforschung und Physik, Universität Salzburg, Hellbrunnerstr. 34, 5020 Salzburg, Austria

**Keywords:** DFT, CASTEP, NaAlSi_3_O_8_, KAlSi_3_O_8_, Vibrational entropy, Infrared spectra, Low microcline, Low albite

## Abstract

The heat capacities of Al, Si ordered alkali feldspars of different Na, K compositions were calculated using the density functional theory. The effect of the Na, K distribution, if random, ordered or clustered, on the resulting heat capacity was investigated on different cells with Ab_50_Or_50_ composition. For all compositions and distributions studied, the excess heat capacity of mixing is positive at low temperatures with a maximum at ~60 K. This produces an increasing excess vibrational entropy that reaches a constant value above ~200 K. The amount of the excess heat capacity of Ab_50_Or_50_, however, depends on the Na, K distribution. Best agreement with measured excess heat capacities is achieved, if the distribution of Na and K is either ordered or clustered. The positive excess heat capacities can be attributed to a strong softening of the acoustic and the lowest optical modes related to a strong increase of Na–O bond lengths in samples with intermediate compositions. The softening of the lowest optical mode is, however, not reflected by thoroughly measured literature IR data. Comparing calculated and measured IR spectra suggests that the resolution of the measured spectra was insufficient for detecting the lowest IR-active modes.

**Electronic supplementary material:**

The online version of this article (doi:10.1007/s00269-014-0715-8) contains supplementary material, which is available to authorized users.

## Introduction

The heat capacity of solid solutions when plotted as a function of composition often deviates from linear behaviour at low temperatures. The resulting excess heat capacities are of vibrational and/or non-lattice (e.g. magnetic and electronic) origin. If the non-lattice contributions are not present or can be neglected, as it is the case with the alkali feldspars, only excess vibrational entropies are produced by the excess heat capacities. Excess vibrational entropies may be in the order of the configurational entropy and may have thus large effects on phase stability calculations (for a review of the vibrational entropy in solid solutions, see e.g. Van de Walle and Ceder 2002; Fultz 2010).

According to the “bond length versus bond stiffness interpretation” of the excess vibrational entropy, changes of the stiffness of a chemical bond are due to accompanied changes of the bond length, which occur during compositional variations (e.g. Van de Walle and Ceder 2002; Wu et al. 2003; Burton and van de Walle 2006; Van de Walle 2009). Assuming a typical value of 2 for the Grüneisen parameter Fultz (2010) estimated that a bond length increase of only 1 % decreases the force constant by 12 %, which is a large softening effect. A bond length increase of 10 % drops the force constant to zero. Bond softening increases the vibrational entropy because frequencies of lattice vibrations are shifted to lower values causing an increase in heat capacity and thus entropy, whereas bond stiffening has the opposite effect (Van de Walle and Ceder 2002). Mutual substitution of atoms of different size generally results in an increase in bond lengths around the smaller atom (bond softening) and a corresponding decrease around the larger atom (bond stiffening) compared to the bond lengths of the respective end-member structures. One of the both effects, however, may dominate the vibrational behaviour in the solid solution resulting in positive or negative excess vibrational entropies. Assuming a binary solid solution with end-members A and B, which have different volumes and elastic constants, it is to be expected that with compositional variation the bond length change of the stiffer end-member is less pronounced compared to that of the softer (Benisek and Dachs 2011). Under the assumption that the larger end-member is elastically stiffer, the atoms of the smaller end-member have to enlarge their bond lengths to a high degree, producing positive excess vibrational entropies as a net effect. The opposite situation may produce negative excess vibrational entropies, if the difference in elasticity is large enough. These considerations lead to an empirical relationship (Benisek and Dachs 2011, 2012) allowing the estimation of the maximum extent of the excess vibrational entropy, Δ_max_ *S* ^exc^, from the differences of end-member volumes, Δ*V* = *V*_A_–*V*_B_, and bulk moduli, Δ*K* = *K*_A_–*K*_B_:

1 $$\Delta_{\hbox{max} } S^{\text{exc}} = (\Delta V + {\text{m}}\;\Delta K){\text{f}}$$

The value of Δ*V* is defined to be positive so that Δ*K* may be positive or negative depending on which end-member is elastically stiffer (smaller or larger) distinguishing the above-mentioned cases. The parameters m and f were determined based on data of some silicate solid solutions and binary alloys (Benisek and Dachs 2012). In the following, this Δ*V* versus Δ*K* approach was applied to other binary solid solution systems as well (Benisek and Dachs 2013, Eremin et al. 2013, Benisek et al. 2013, 2014a, b; Dachs et al. 2014a, b).

First-principles methods enable nowadays to calculate the effects of the vibrational modes in solid solutions producing more and more realistic phase stability diagrams (e.g. Turchi et al. 1991; Tepesch et al. 1996; Burton and van de Walle 2006; Urusov et al. 2007; Vinograd and Winkler 2010). Using the density functional theory (DFT), the lattice vibrations of the low albite—low microcline series, i.e. Al, Si ordered alkali feldspars in the system NaAlSi_3_O_8_–KAlSi_3_O_8_ abbreviated with Ab and Or are modelled in this study. The frequencies of the acoustic and optical modes are plotted as a function of composition in order to identify those vibrational modes that are most affected within the solid solution. Low-frequency modes are to be expected to be strongly softened in samples with intermediate compositions since alkali feldspars are characterised by distinct positive excess heat capacities at about 80 K (Benisek et al. 2014a). Such behaviour, however, is not reflected by the measured infrared (IR) spectra of the alkali feldspars (Zhang et al. 1996). Although the spectra in the low-wavenumber region are not easy to interpret, the lowest frequency mode measured experimentally is shifted to slightly higher frequencies in samples with intermediate composition, which is at variance with expectations from the heat capacity data.

## Computational methods

Quantum–mechanical calculations were based on the DFT plane wave pseudopotential approach implemented in the CASTEP code (Clark et al. 2005) included in the Materials Studio software from Accelrys^®^. The calculations used the local density approximation for the exchange–correlation functional (Ceperley and Alder 1980) and norm-conserving pseudopotentials to describe the core-valence interactions. The 2s^2^2p^6^3s^1^, 3s^2^3p^6^4s^1^, 3s^2^3p^1^, 3s^2^3p^2^ and 2s^2^2p^4^ electrons were explicitly treated as valence electrons for Na, K, Al, Si and O, respectively. For the k-point sampling of the investigated single unit cells, a 2 × 1 × 2 Monkhorst–Pack grid was used (Monkhorst and Pack 1976) and convergence was tested by performing phonon calculations using a denser k-point grid. The structural relaxation was calculated applying the BFGS algorithm, where the threshold for the force on the atom was 0.01 eV Å^−1^. The lattice dynamical calculations were performed for these relaxed structures within the linear response approximation implemented in CASTEP using the interpolation approach. DFT parameter settings used in the calculations are listed in Table 1.

**Table 1:** DFT parameter settings

Cut-off for plane wave basis set	830 eV
Grid for fast Fourier transform	80 × 120 × 72
Convergence threshold for self-consistent field	5 × 10^−7^ eV atom^−1^
Spacing for *k*-point sampling^a^	~0.07 Å^−1^
Spacing for *q*-point sampling	~0.05 Å^−1^

^a^Convergence was investigated using an increasingly dense *k*-point mesh

## Structural models

All investigated cells are characterised by a fully ordered Al, Si distribution, i.e. Al occupies the T_1_O-site and Si the other three tetrahedral sites. The end-member structures of albite and microcline have a triclinic symmetry ($\bar{1}$), whereas in cells with intermediate compositions, the Na–K substitution destroys the symmetry elements. This is different to a real alkali feldspar crystal, where the Na and K sites are thermodynamically averaged. To investigate different Na, K configurations, four cells with Ab_50_Or_50_ composition were constructed: two ordered configurations using the single unit cell (cell 1 and 2) containing 4 A-sites (occupied either by Na or K) and two supercells containing 16 A-sites. For one of the supercells, a random number generator of Mathematica^®^ constructed a quasi-random Na, K distribution (cell 3) and the other supercell was used to simulate a clustered Na, K distribution (cell 4). The clusters represent thin (up to 2 atoms) irregularly shaped lamellae in the (100)-plane. For the other compositions (Ab_75_Or_25_ and Ab_25_Or_75_), single unit cells were used, where only one A-site was substituted by the other atom. Appendix A (available as electronic supplementary material) contains the crystal symmetry, the lattice parameters, the fractional coordinates, the enthalpies and plots of the relaxed structures of all investigated cells. The calculated volumes are smaller by ~5 % compared to measured volumes. The calculated excess volumes of mixing, however, agree well with measured ones, i.e. they lie in the middle of the range defined by the results of Kroll et al. (1986), Hovis and Peckins (1978), Hovis (1988) as shown in Appendix A.

## Results

**Fig. 1:**
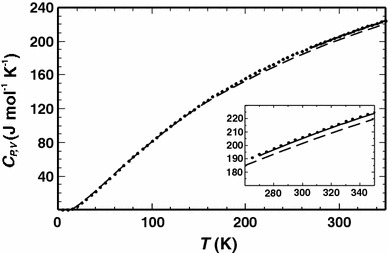
Heat capacities of low albite plotted against temperature. *Points* measured isobaric heat capacity (*C*_*P*_) from Benisek et al. (2014a); *Broken line* calculated isochoric heat capacity (*C*_*V*_); in the temperature region between 270 and 350 K, *C*_*V*_ was transformed into *C*_*P*_ (*solid line*)

**Fig. 2:**
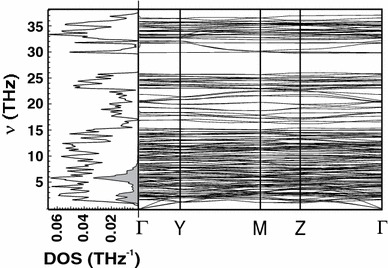
Phonon dispersion relation (frequency, *ν*, versus wave vector) and phonon DOS of low albite. Partial phonon DOS of the Na atoms is given by the shaded area. *Γ* represents the centre, whereas *Y*, *M* and *Z* are points at the edges of the Brillouin zone with the coordinates (0, ½, 0), (0, ½, ½) and (0, 0, ½), respectively

In the first step, the heat capacities of the end-members with ordered Al, Si distribution were calculated. The results for low albite are compared with measured ones in Fig. 1. In the temperature range of 270–350 K, the calculated isochoric heat capacity (*C*_*V*_) was transformed into the isobaric heat capacity (*C*_*P*_), using the relationship *C*_*P*_ = *C*_*V*_ + *T* *V* α^2^ *K*, where the volume, *V*, the thermal-expansion coefficient, *α*, and the bulk modulus, *K*, were taken from the literature (Kroll and Ribbe 1983; Tribaudino et al. 2010; Downs et al. 1994). The calculated *C*_*P*_ is in good agreement with the measured data with a deviation of less than 0.4 %. The calculated phonon dispersion relation and the phonon density of states (DOS) for low albite are displayed in Fig. 2. The frequencies of the acoustic modes range from zero to ca. 1.2 THz at the edge of the Brillouin zone. The optical mode frequencies range from 1.9 to 37 THz (63–1,233 cm^−1^) with a gap between 26 and 29 THz. A partial phonon DOS of the Na atoms is also shown depicting the large contribution of the Na vibrations to the phonon DOS at low frequencies.

**Fig. 3:**
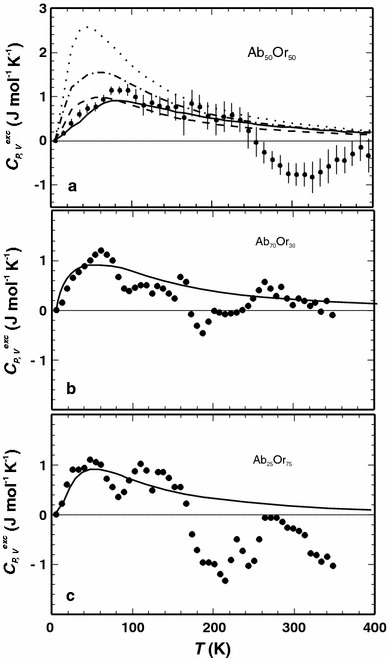
Excess heat capacities (*C*_*P, V*_^exc^) plotted against temperature (*T*). *Points* measurements from Benisek et al. (2014a), *lines* represent the DFT results. **a** Data of Ab_50_Or_50_ of cell 1 (*solid*), cell 2 (*dashed*-*dotted*), cell 3 (*dotted*) and cell 4 (*dashed*). **b** Measured data of Ab_70_Or_30_, calculated ones of Ab_75_Or_25_. **c** Data of Ab_25_Or_75_. *Error bars* as shown in **a** represent 1 SD

In the second step, the heat capacities of the solid solutions with intermediate compositions (Ab_75_Or_25_, Ab_50_Or_50_ and Ab_25_Or_75_) were calculated. The calculated heat capacities of all solid solution compositions and cells deviate positively from the linear combination of the end-member heat capacities (i.e. *C*_*V*_^linear^ = *X*_A_ *C*_*V*_^A^ + *X*_B_ *C*_*V*_^B^) at low temperatures. The behaviour of the resulting excess heat capacity as a function of temperature is characterised by a distinct positive peak at ~60 K for all cells. The calculated excess heat capacities (*C*_*V*_^exc^) of Ab_50_Or_50_ are compared with measured ones (*C*_*P*_^exc^) in Fig. 3a. The structure with a quasi-random Na, K distribution (cell 3) results in large excess heat capacities, whereas the other configurations agree more or less with the measured data. The calculated excess heat capacities do not exhibit the negative peak of the measured data at ~320 K. This negative peak, however, may not be significant taking into account the uncertainty of the experimental data (error bars in Fig. 3a represent one standard deviation). The calculated excess heat capacities of Ab_75_Or_25_ and Ab_25_Or_75_ are in accordance with the calorimetrically derived values as shown in Fig. 3b, c.

**Fig. 4:**
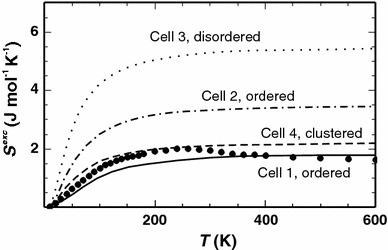
The excess vibrational entropy (*S* ^exc^) of Ab_50_Or_50_ plotted against temperature. *Data points* represent the calorimetric results of Benisek et al. (2014a) and the *lines* those of the DFT calculations of cell 1 (*solid*), cell 2 (*dashed*-*dotted*), cell 3 (*dotted*) and cell 4 (*dashed*)

Figure 4 depicts the results for the excess vibrational entropy of Ab_50_Or_50_ as a function of temperature demonstrating that the excess vibrational entropy of all investigated configurations becomes constant above approximately 200 K. However, the excess vibrational entropy of cells 2 and 3 (ordered and disordered Na, K distribution) is larger than the measured data. Further results presented below for Ab_50_Or_50_ were, therefore, taken from calculations using cell 1 (ordered Na, K distribution) because of its good agreement with the calorimetric data. For more details, concerning the influence of the Na, K configurations on the mode frequency, see Appendix B (available as electronic supplementary material), where the calculated dispersion relations in the low-frequency range of the end-members and the different Ab_50_Or_50_ cells are compared. This appendix also shows the Brillouin zone including symmetry points and their coordinates.

**Fig. 5:**
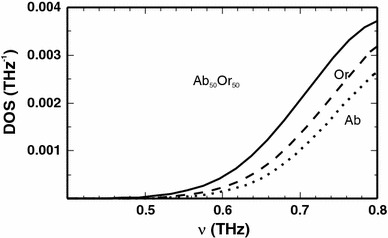
Low-frequency part of the phonon DOS for low albite (*dotted line*), low microcline (*dashed line*) and low structural state Ab_50_Or_50_ of cell 1 (*solid line*)

**Fig. 6:**
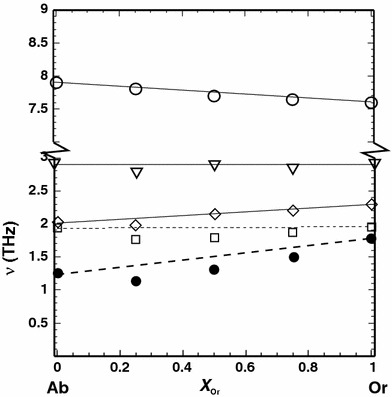
Mean frequencies (*ν*) of vibrational modes in the low-frequency region plotted against composition (*X*_Or_). *Solid circles* Acoustic modes at the edge of the Brillouin zone (Z); *Open squares* the five lowest optical modes at the centre of the Brillouin zone (Γ); *Open diamonds* the same five optical modes at point Z; *Open triangles* three optical modes at the centre of the Brillouin zone at ~3 THz; *Open circles* an optical mode at ~8 THz. The *lines* represent the behaviour of a linear combination of the end-member frequencies

In Fig. 5, the low cut-off frequency of the phonon DOS for the two end-members and for the Ab_50_Or_50_ composition is shown, demonstrating the shift to lower frequencies in the Ab_50_Or_50_ sample, which is the reason for the positive excess heat capacity at low temperatures. The frequencies of the vibrational modes responsible for this shift are separately plotted as a function of composition in Fig. 6. As can be seen, the frequencies of the acoustic modes at the edge of the Brillouin zone (at point Z as an example) are mostly affected by the substitution producing a distinct mode softening in samples with intermediate compositions. Using a linear combination of the end-member frequencies of these modes results in 1.5 THz at *X*_Or_ = 0.5, whereas the solid solution with this composition is characterised by a mean acoustic mode frequency of only 1.3 THz. The lowest optical modes are also affected by the substitution (down shift by ~0.1 THz). At slightly higher frequencies (~3 and 8 THz), the optical modes exhibit an almost ideal mixing behaviour (Fig. 6).

## Discussion

**Fig. 7:**
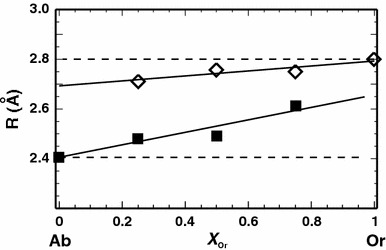
Mean bond lengths (R) of Na–O (*closed symbols*) and K–O (*open symbols*) as a function of composition. *Solid lines* represent a fit through the data, whereas the *broken lines* show the bond lengths without changes across the solid solution

The positive excess heat capacities and the correspondingly positive excess vibrational entropies are caused by the large increase of Na–O bond lengths in samples with intermediate compositions. This behaviour can be derived from Fig. 7 and Table 2, where the Na–O and K–O bond length changes due to compositional changes are presented. The mean bond length of Na–O increases by 3.7 %, whereas that of K–O decreases by only 1.5 % when changing the composition from the respective end-members to Ab_50_Or_50_. Detailed information of the Na–O and K–O bond lengths is listed as a function of composition in Appendix C (available as electronic supplementary material).

**Table 2:** Mean DFT calculated Na, K–O bond lengths (R) in the respective end-members and their relative changes (Δ) due to compositional changes (from end-member to Ab_50_Or_50_ composition). Na and K are coordinated fivefold and sevenfold, respectively, which is in accordance with Downs et al. (1996)

	R (Å)	Δ (%)
Na–O	2.41	+3.7
K–O	2.80	−1.5

**Fig. 8:**
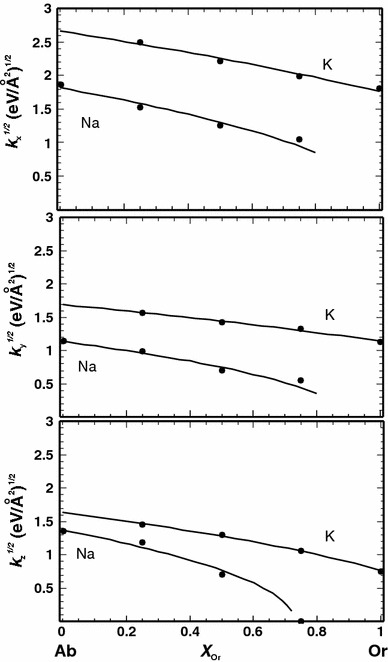
Square root of the force constants in *x* , *y* and *z* direction (*k*_x_, *k*_y_ and *k*_z_) of sodium and potassium as a function of composition (*X*_Or_). The curves are based on a linear fit of *k* versus *X*_Or_

The force constants, *k*, of sodium and potassium, i.e. the diagonal elements, $k = \frac{{\partial^{2} V}}{{\partial x^{2} }}$, of the force constant matrix behave differently (where *V* and x are the potential energy and direction, respectively). The component of the force constant in x direction, e.g. decreases by 58 % for sodium, whereas that of potassium increases by 52 % when going from the respective end-members to Ab_50_Or_50_. The extent of the changes of both force constant values is thus similar. The heat capacity behaviour, however, depends on the phonon frequencies (*ν*). Because of the proportionality *ν* ~ *k* ^1/2^, the influence of a force constant increase on frequencies is smaller than a force constant decrease as demonstrated in Fig. 8, where the square root of the *k* values of sodium and potassium is plotted against composition. In all three directions, *k* ^1/2^ of sodium decreases strongly with increasing potassium content and this decrease has a steeper slope than the corresponding line representing *k* ^1/2^ for potassium. The strongest decrease of *k* ^1/2^ for sodium is in z direction, where it drops to zero at *X*_Or_ = 0.75. Similar behaviour was also found for the NaCl–KCl and MgO–CaO solid solution (Burton and Van de Walle 2006).

The structural behaviour of the alkali feldspar solid solution, i.e. the large increase of Na–O bond lengths, can be interpreted from a macroscopic point of view using the Δ*V* versus Δ*K* approach (Benisek and Dachs 2011, 2012). Both the volume and the bulk modulus of low microcline are larger compared to those of low albite (Table 3). Accordingly, the larger and stiffer K-feldspar structure forces the Na–O bonds to enlarge their distances to a high degree. The corresponding phonons are, therefore, strongly softened resulting in the positive excess heat capacities and positive excess vibrational entropies. As shown by Benisek et al. (2014a), the excess vibrational entropy of the Al, Si ordered alkali feldspar solid solution calculated by the Δ*V* versus Δ*K* approach (Eq. 1) agrees well with the calorimetric data.

**Table 3:** Volumes (*V*) and bulk moduli (*K*) of low albite (Ab) and low microcline (Or)

	*V* (J bar^−1^)	*K* (GPa)
Ab	9.994^a^	53.0^c^
Or	10.878^a^	58.3^b^

^a^Kroll and Ribbe (1983)

^b^Allan and Angel (1997)

^c^Downs et al. (1994)

**Fig. 9:**
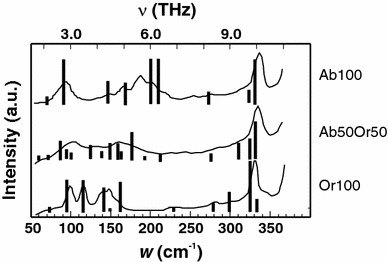
IR spectra in the low-frequency region. The abscissa is shown in units of wavenumber (*w*) as well as frequency (*ν*). *Solid line* measured by Zhang et al. (1996); *Vertical bars* DFT calculations

On the other hand, IR spectra (Zhang et al. 1996) do not exhibit any phonon softening of the lowest frequency mode, which is visible in the IR spectra at ~100 and ~90 cm^−1^ for low microcline and low albite, respectively (Zhang et al. 1996, their Fig. 1). From their figure, a slight shift to even higher frequencies in samples with intermediate composition may be derived suggesting a small stiffening by the substitution process at variance with the positive excess heat capacities measured by calorimetry (Benisek et al. 2014a) and derived from the DFT calculations of this study. In order to resolve this discrepancy, we used the DFT methods to calculate the IR spectra of the end-member and Ab_50_Or_50_ compositions. According to these calculations, the lowest IR-active modes have low intensities and low wavenumbers of 71, 73, and 60 cm^−1^ for Ab, Or and Ab_50_Or_50_ compositions, respectively. Note the softening (by 12 cm^−1^) of this mode with the Na–K substitution. In Fig. 9, the measured low-frequency IR spectra of Zhang et al. (1996) are compared to the calculated IR modes. Some calculated modes have slightly different frequencies, however, in general, good agreement is observed. Even the lowest IR-active mode at 60 cm^−1^ of the Ab_50_Or_50_ sample, as calculated by the DFT methods of this study, might be identified in the observed data by a small shoulder. The resolution of the measured IR data, however, is not sufficient to assign an IR band unequivocally to these low frequencies.

As shown in Figs. 3 and 4, the DFT results obtained for Na, K ordered or clustered distributions yield better agreement with the calorimetric results than the cell with the disordered Na, K distribution. This observation is consistent with investigations on Al, Si ordered alkali feldspars by means of ^23^Na NMR spectroscopy (Phillips et al. 1988). The respective samples were synthesised in the same way and at the same conditions as the samples used for determining the excess heat capacities calorimetrically. The NMR data of these samples indicate short-range clustering, i.e. the Na, K environment of a Na atom is skewed towards a relative enrichment of Na atoms (Phillips et al. 1988) supporting the DFT results.

## Conclusions

Calculations of the phonon spectra and the heat capacity by density functional methods supply a consistent picture combining findings from different studies, viz. from calorimetric and NMR data, as well as from a macroscopic description of the excess vibrational entropy (Δ*V* vs. Δ*K* approach). Furthermore, the calculations succeeded in resolving contradicting results derived from calorimetry and IR spectroscopy.

## Electronic supplementary material

Below is the link to the electronic supplementary material.

Supplementary material 1 (PDF 582 kb)

Supplementary material 2 (PDF 1476 kb)

Supplementary material 3 (PDF 11 kb)
