# Supplementary material for: First-principles investigation of the lattice vibrations in the alkali feldspar solid solution
Source: Phys Chem Miner. 2014 Oct 11;42(3):243–9. doi: 10.1007/s00269-014-0715-8 (PMC4509561; doi:10.1007/s00269-014-0715-8)
Supplement: Supplementary file 1 — Supplementary material 1 (PDF 582 kb) [file 269_2014_715_MOESM1_ESM.pdf]

Appendix A:
Structural details of all investigated cells of the study
"First-principles investigation of the lattice vibrations in the alkali feldspar solid solution" published by Artur Benisek, Edgar Dachs, Michael Grodzicki in Physics and Chemistry of Minerals.

xxxxxxxxxxxxxxxxxxxxxxxxxxxxxxxxxxxxxxxxxxxxxxxxxxxxxxxxxxxxxxxxxxxxxxxxx
Ab100, Low albite

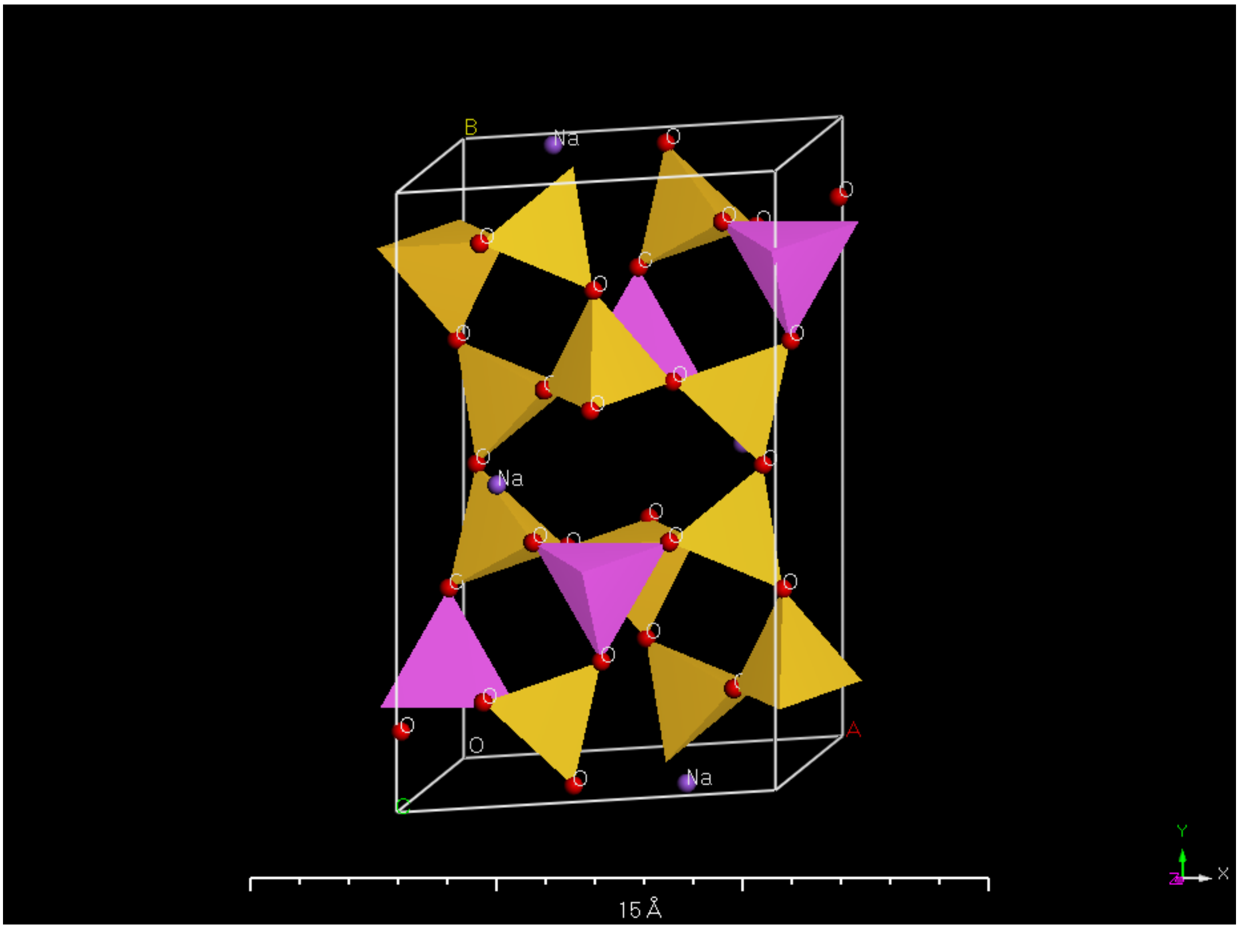

(Al-tetrahedron: magenta, Si-tetrahedron: yellow)

```
                 Number of symmetry operations   =           4
                       Point group of crystal =     2: Ci, -1, -1

                      Lattice parameters(A)        Cell Angles
                    a =     7.995950           alpha =    94.186669
                    b =    12.619333           beta  =   117.039304
                    c =     7.062478           gamma =    88.108769

                Current cell volume =  633.041346 A**3

              BFGS: Final Enthalpy     = -1.99962241E+004 eV
```

x	Element	Atom Number	Fractional coordinates of atoms u	v	w	x
x	O	1	0.005608	0.127522	0.970426	x
x	O	2	0.582265	0.995995	0.275644	x

x	O	3	0.813815	0.105014	0.189395	x
x	O	4	0.817185	0.851741	0.255890	x
x	O	5	0.007926	0.297594	0.267765	x
x	O	6	0.019430	0.693673	0.218254	x
x	O	7	0.208520	0.108728	0.388244	x
x	O	8	0.187946	0.867740	0.437259	x
x	O	9	0.505608	0.627522	0.970426	x
x	O	10	1.082265	1.495995	0.275644	x
x	O	11	1.313815	0.605014	0.189395	x
x	O	12	1.317185	1.351741	0.255890	x
x	O	13	0.507926	0.797594	0.267765	x
x	O	14	0.519430	1.193673	0.218254	x
x	O	15	0.708520	0.608728	0.388244	x
x	O	16	0.687946	1.367740	0.437259	x
x	O	17	-0.005608	-0.127522	-0.970426	x
x	O	18	-0.582265	-0.995995	-0.275644	x
x	O	19	-0.813815	-0.105014	-0.189395	x
x	O	20	-0.817185	-0.851741	-0.255890	x
x	O	21	-0.007926	-0.297594	-0.267765	x
x	O	22	-0.019430	-0.693673	-0.218254	x
x	O	23	-0.208520	-0.108728	-0.388244	x
x	O	24	-0.187946	-0.867740	-0.437259	x
x	O	25	0.494392	0.372478	-0.970426	x
x	O	26	-0.082265	-0.495995	-0.275644	x
x	O	27	-0.313815	0.394986	-0.189395	x
x	O	28	-0.317185	-0.351741	-0.255890	x
x	O	29	0.492074	0.202406	-0.267765	x
x	O	30	0.480570	-0.193673	-0.218254	x
x	O	31	0.291480	0.391272	-0.388244	x
x	O	32	0.312054	-0.367740	-0.437259	x
x	Na	1	0.260503	0.992376	0.142319	x
x	Na	2	0.760503	1.492376	0.142319	x
x	Na	3	-0.260503	-0.992376	-0.142319	x
x	Na	4	0.239497	-0.492376	-0.142319	x
x	Al	1	0.008184	0.165261	0.208268	x
x	Al	2	0.508184	0.665261	0.208268	x
x	Al	3	-0.008184	-0.165261	-0.208268	x
x	Al	4	0.491816	0.334739	-0.208268	x
x	Si	1	0.003005	0.821544	0.233708	x
x	Si	2	0.687611	0.108786	0.309894	x
x	Si	3	0.675877	0.880458	0.358238	x
x	Si	4	0.503005	1.321544	0.233708	x
x	Si	5	1.187611	0.608786	0.309894	x
x	Si	6	1.175877	1.380458	0.358238	x
x	Si	7	-0.003005	-0.821544	-0.233708	x
x	Si	8	-0.687611	-0.108786	-0.309894	x
x	Si	9	-0.675877	-0.880458	-0.358238	x
x	Si	10	0.496995	-0.321544	-0.233708	x
x	Si	11	-0.187611	0.391214	-0.309894	x
x	Si	12	-0.175877	-0.380458	-0.358238	x

xxxxxxxxxxxxxxxxxxxxxxxxxxxxxxxxxxxxxxxxxxxxxxxxxxxxxxxxxxxxxxxxxxxxxxxxxx

Ab75Or25

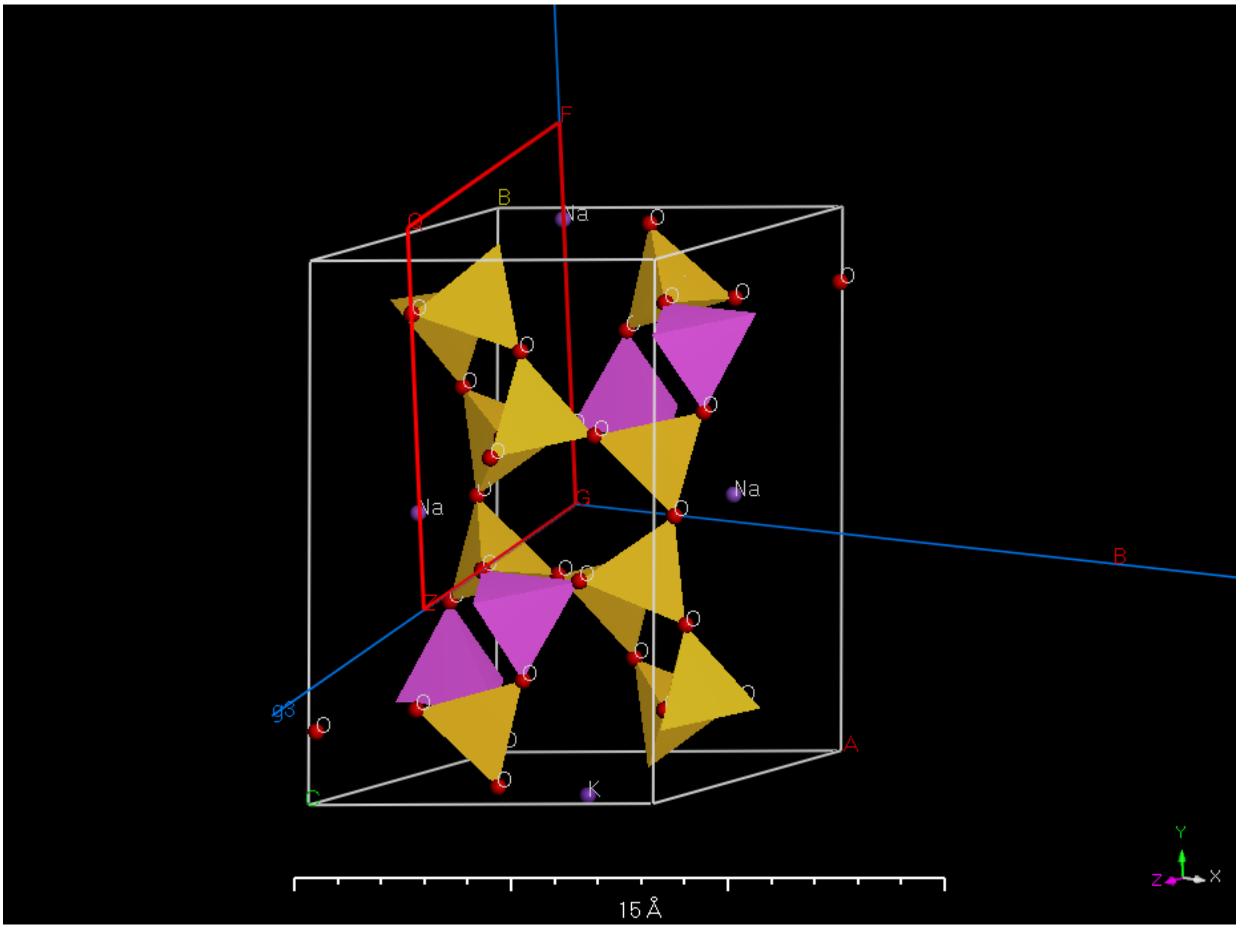

There are no symmetry operations specified or generated for this cell

	Lattice parameters(A)		Cell Angles
a =	8.113125	alpha =	93.741534
b =	12.679461	beta =	116.647441
c =	7.081416	gamma =	88.022166

Current cell volume = 649.691356 A**3

BFGS: Final Enthalpy = -1.96263049E+004 eV

Element	Atom Number	Fractional coordinates of atoms u	v	w
O	1	0.011279	0.132478	0.983025
O	2	0.593538	0.996578	0.281651
O	3	0.826617	0.114598	0.219052
O	4	0.811049	0.853850	0.226376
O	5	0.012761	0.303216	0.274110
O	6	0.023265	0.692643	0.228614
O	7	0.211892	0.113986	0.390078
O	8	0.176721	0.869798	0.430560
O	9	0.505443	0.633114	0.966187
O	10	0.092095	0.497769	0.278296
O	11	0.313895	0.611824	0.185969
O	12	0.321169	0.350300	0.263909
O	13	0.517258	0.798958	0.263892

x	O	14	0.523003	0.193526	0.228417	x
x	O	15	0.703299	0.607435	0.384320	x
x	O	16	0.684462	0.367307	0.438363	x
x	O	17	1.005372	0.863199	0.023123	x
x	O	18	0.395756	0.004768	0.718633	x
x	O	19	0.194285	0.883190	0.813927	x
x	O	20	0.165359	0.148453	0.733037	x
x	O	21	0.993290	0.692974	0.725238	x
x	O	22	0.972452	0.306488	0.780179	x
x	O	23	0.810131	0.881718	0.597228	x
x	O	24	0.802905	0.131562	0.580033	x
x	O	25	0.494231	0.372200	0.034636	x
x	O	26	0.907049	0.501666	0.721384	x
x	O	27	0.680134	0.391054	0.810127	x
x	O	28	0.684824	0.651693	0.742373	x
x	O	29	0.479964	0.202266	0.742811	x
x	O	30	0.478780	0.808518	0.759555	x
x	O	31	0.292701	0.393507	0.616200	x
x	O	32	0.319918	0.631581	0.562039	x
x	Na	1	0.264581	0.991324	0.141634	x
x	Na	2	0.767491	0.485257	0.150758	x
x	Na	3	0.229450	0.520026	0.843252	x
x	Al	1	0.012776	0.171225	0.218466	x
x	Al	2	0.509497	0.667072	0.204848	x
x	Al	3	0.999763	0.824701	0.785999	x
x	Al	4	0.487037	0.334737	0.795837	x
x	Si	1	0.002927	0.819415	0.226651	x
x	Si	2	0.692558	0.111288	0.325959	x
x	Si	3	0.684700	0.878997	0.348401	x
x	Si	4	0.503068	0.320865	0.238302	x
x	Si	5	0.193376	0.610305	0.311286	x
x	Si	6	0.181608	0.382511	0.364042	x
x	Si	7	0.990222	0.179621	0.770832	x
x	Si	8	0.305757	0.889357	0.680802	x
x	Si	9	0.313080	0.121595	0.642024	x
x	Si	10	0.499439	0.681094	0.760898	x
x	Si	11	0.804790	0.389612	0.689590	x
x	Si	12	0.819320	0.617023	0.636133	x
x	K	1	0.733663	0.001760	0.860945	x

xxxxxxxxxxxxxxxxxxxxxxxxxxxxxxxxxxxxxxxxxxxxxxxxxxxxxxxxxxxxxxxxxxxxxxxxxxxxxxx

Ab50Or50, cell 1

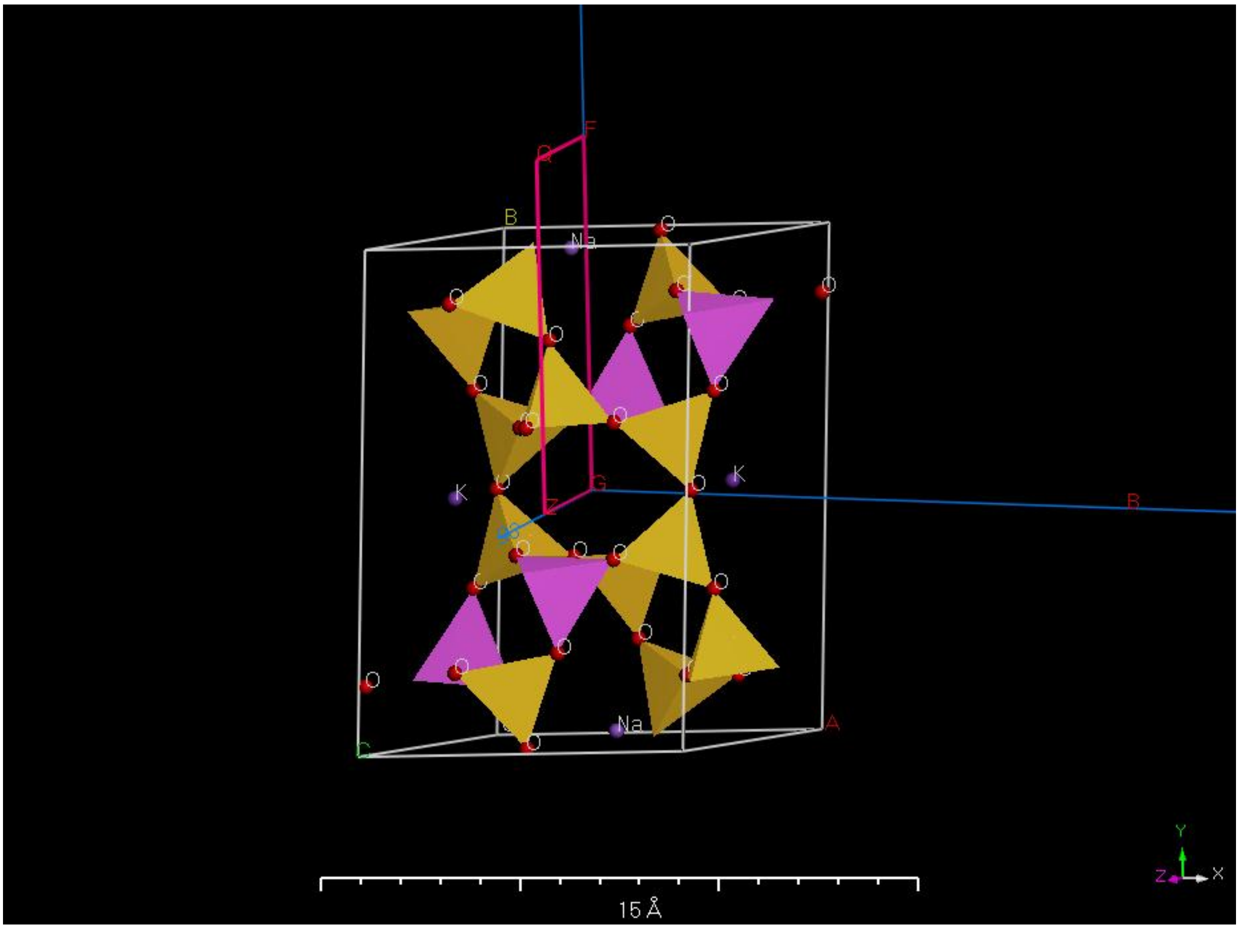

There are no symmetry operations specified or generated for this cell

Lattice parameters(A)		Cell Angles	
a =	8.186195	alpha =	93.201511
b =	12.750190	beta =	115.705142
c =	7.095594	gamma =	88.082983

Current cell volume = 666.238082 A**3

BFGS: Final Enthalpy = -1.92566312E+004 eV

Element	Atom Number	Fractional coordinates of atoms u	v	w
O	1	0.001840	0.137059	0.958910
O	2	0.603771	1.001019	0.287524
O	3	0.819141	0.117391	0.188015
O	4	0.820037	0.848576	0.268809
O	5	0.030762	0.299139	0.256184
O	6	0.019865	0.689855	0.254416
O	7	0.201581	0.104690	0.376480
O	8	0.178465	0.866012	0.437375
O	9	0.497362	0.642761	0.990901
O	10	0.111572	0.496071	0.290605
O	11	0.316226	0.628319	0.220500
O	12	0.330074	0.358475	0.238094
O	13	0.512597	0.813342	0.285551

x	O	14	0.530827	0.194069	0.235163	x
x	O	15	0.693269	0.623273	0.402083	x
x	O	16	0.690506	0.371693	0.409667	x
x	O	17	0.998160	0.862941	0.041090	x
x	O	18	0.396229	-0.001019	0.712476	x
x	O	19	0.180859	0.882609	0.811985	x
x	O	20	0.179963	0.151424	0.731191	x
x	O	21	0.969239	0.700861	0.743816	x
x	O	22	0.980135	0.310145	0.745584	x
x	O	23	0.798419	0.895310	0.623520	x
x	O	24	0.821535	0.133988	0.562625	x
x	O	25	0.502639	0.357239	0.009099	x
x	O	26	0.888428	0.503929	0.709395	x
x	O	27	0.683774	0.371681	0.779500	x
x	O	28	0.669926	0.641525	0.761906	x
x	O	29	0.487403	0.186659	0.714449	x
x	O	30	0.469173	0.805931	0.764837	x
x	O	31	0.306731	0.376727	0.597917	x
x	O	32	0.309494	0.628307	0.590333	x
x	Na	1	0.281254	0.962838	0.175189	x
x	Na	2	0.718746	0.037162	0.824811	x
x	Al	1	0.014541	0.167803	0.200872	x
x	Al	2	0.504112	0.682178	0.226368	x
x	Al	3	0.985459	0.832197	0.799128	x
x	Al	4	0.495888	0.317822	0.773632	x
x	Si	1	0.000658	0.816689	0.245449	x
x	Si	2	0.700016	0.113322	0.314539	x
x	Si	3	0.687428	0.886065	0.374236	x
x	Si	4	0.511965	0.319642	0.222373	x
x	Si	5	0.195524	0.612825	0.339236	x
x	Si	6	0.195657	0.378100	0.351141	x
x	Si	7	0.999342	0.183311	0.754551	x
x	Si	8	0.299984	0.886678	0.685461	x
x	Si	9	0.312572	0.113935	0.625764	x
x	Si	10	0.488035	0.680358	0.777627	x
x	Si	11	0.804476	0.387175	0.660764	x
x	Si	12	0.804343	0.621900	0.648859	x
x	K	1	0.768163	0.499402	0.128675	x
x	K	2	0.231837	0.500598	0.871326	x

xxxxxxxxxxxxxxxxxxxxxxxxxxxxxxxxxxxxxxxxxxxxxxxxxxxxxxxxxxxxxxxxxxxxxxxxxxxxxxxxxxxxxxxxxxxxxxxxxxxx

Ab50Or50, cell 2

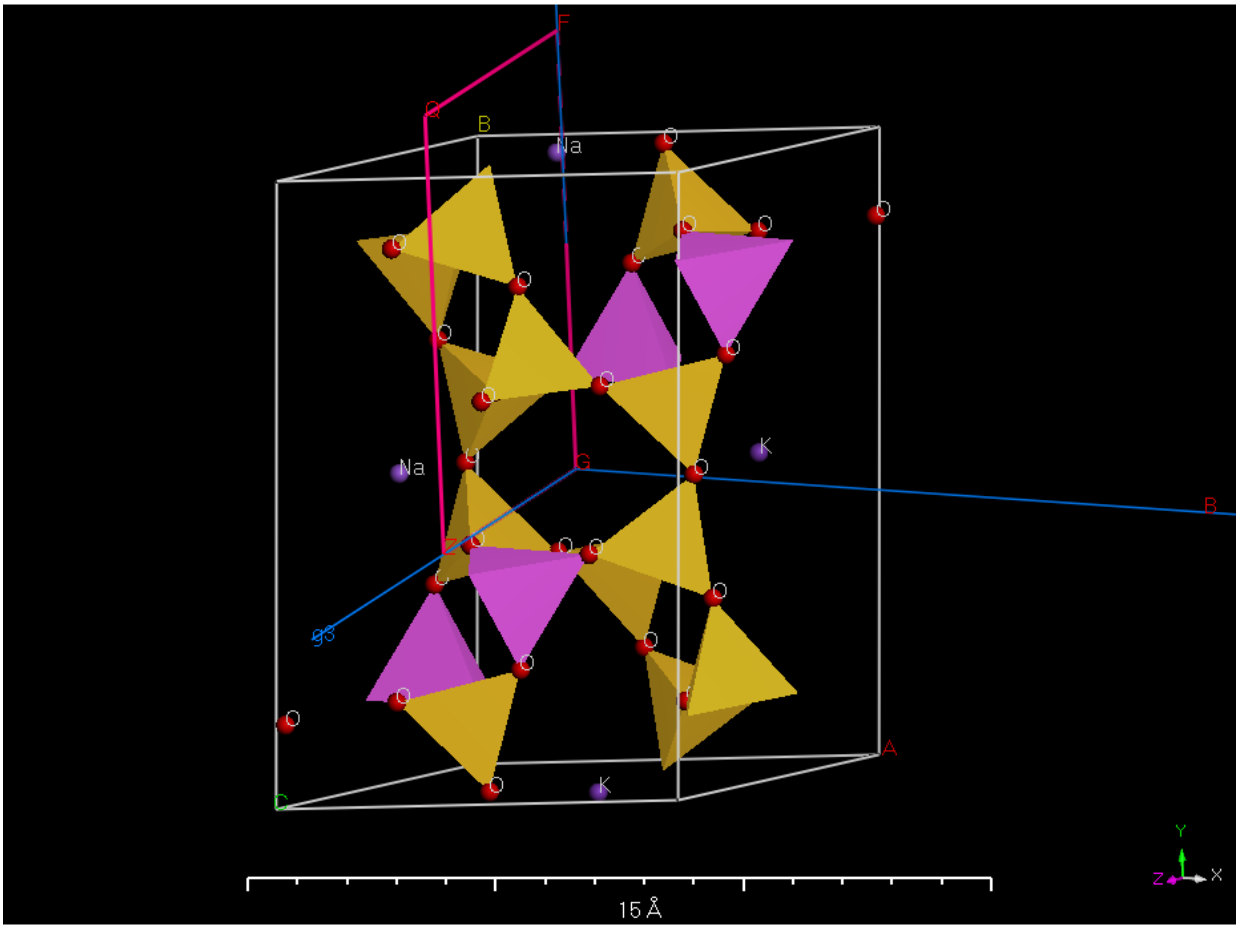

There are no symmetry operations specified or generated for this cell

Lattice parameters (A)		Cell Angles	
a =	8.214162	alpha =	92.860659
b =	12.737500	beta =	116.380025
c =	7.100450	gamma =	87.821991

Current cell volume = 664.610560 A**3

BFGS: Final Enthalpy = -1.92565566E+004 eV

x	Element	Atom Number	Fractional coordinates of atoms u	v	w	x
x	O	1	0.012077	0.132142	0.979892	x
x	O	2	0.603475	0.999933	0.283285	x
x	O	3	0.831886	0.119535	0.219265	x
x	O	4	0.812398	0.852983	0.231173	x
x	O	5	0.023632	0.303849	0.261248	x
x	O	6	0.026059	0.692694	0.249767	x
x	O	7	0.211246	0.111955	0.388304	x
x	O	8	0.173592	0.871702	0.428016	x
x	O	9	0.496864	0.639865	0.975206	x
x	O	10	0.110184	0.498395	0.281216	x
x	O	11	0.309467	0.624435	0.190334	x
x	O	12	0.335803	0.352018	0.270928	x
x	O	13	0.514298	0.809160	0.263415	x

x	O	14	0.531057	0.194759	0.232645	x
x	O	15	0.690161	0.619316	0.400256	x
x	O	16	0.691763	0.369656	0.420248	x
x	O	17	1.003136	0.860135	0.024794	x
x	O	18	0.389816	0.001605	0.718784	x
x	O	19	0.190533	0.875565	0.809666	x
x	O	20	0.164197	0.147982	0.729072	x
x	O	21	0.985702	0.690840	0.736585	x
x	O	22	0.968943	0.305241	0.767354	x
x	O	23	0.809840	0.880685	0.599744	x
x	O	24	0.808237	0.130344	0.579752	x
x	O	25	0.487923	0.367857	0.020108	x
x	O	26	0.896524	0.500067	0.716715	x
x	O	27	0.668114	0.380464	0.780735	x
x	O	28	0.687602	0.647018	0.768827	x
x	O	29	0.476368	0.196150	0.738752	x
x	O	30	0.473941	0.807307	0.750233	x
x	O	31	0.288754	0.388045	0.611696	x
x	O	32	0.326408	0.628298	0.571984	x
x	Na	1	0.272298	0.980204	0.153457	x
x	Na	2	0.227702	0.519796	0.846544	x
x	Al	1	0.017394	0.171830	0.214877	x
x	Al	2	0.503129	0.677613	0.211429	x
x	Al	3	0.996871	0.822387	0.788571	x
x	Al	4	0.482606	0.328169	0.785123	x
x	Si	1	0.002289	0.818778	0.231658	x
x	Si	2	0.700269	0.113404	0.327039	x
x	Si	3	0.687958	0.881866	0.352086	x
x	Si	4	0.508807	0.321001	0.232969	x
x	Si	5	0.199071	0.612844	0.322225	x
x	Si	6	0.190346	0.381339	0.361057	x
x	Si	7	0.991193	0.178999	0.767031	x
x	Si	8	0.300929	0.887156	0.677775	x
x	Si	9	0.309654	0.118661	0.638943	x
x	Si	10	0.497711	0.681222	0.768343	x
x	Si	11	0.799731	0.386596	0.672961	x
x	Si	12	0.812042	0.618134	0.647914	x
x	K	1	0.770006	0.494326	0.140841	x
x	K	2	0.729994	0.005674	0.859159	x

xxxxxxxxxxxxxxxxxxxxxxxxxxxxxxxxxxxxxxxxxxxxxxxxxxxxxxxxxxxxxxxxxxxxxx

Ab50Or50, cell 3 (supercell)

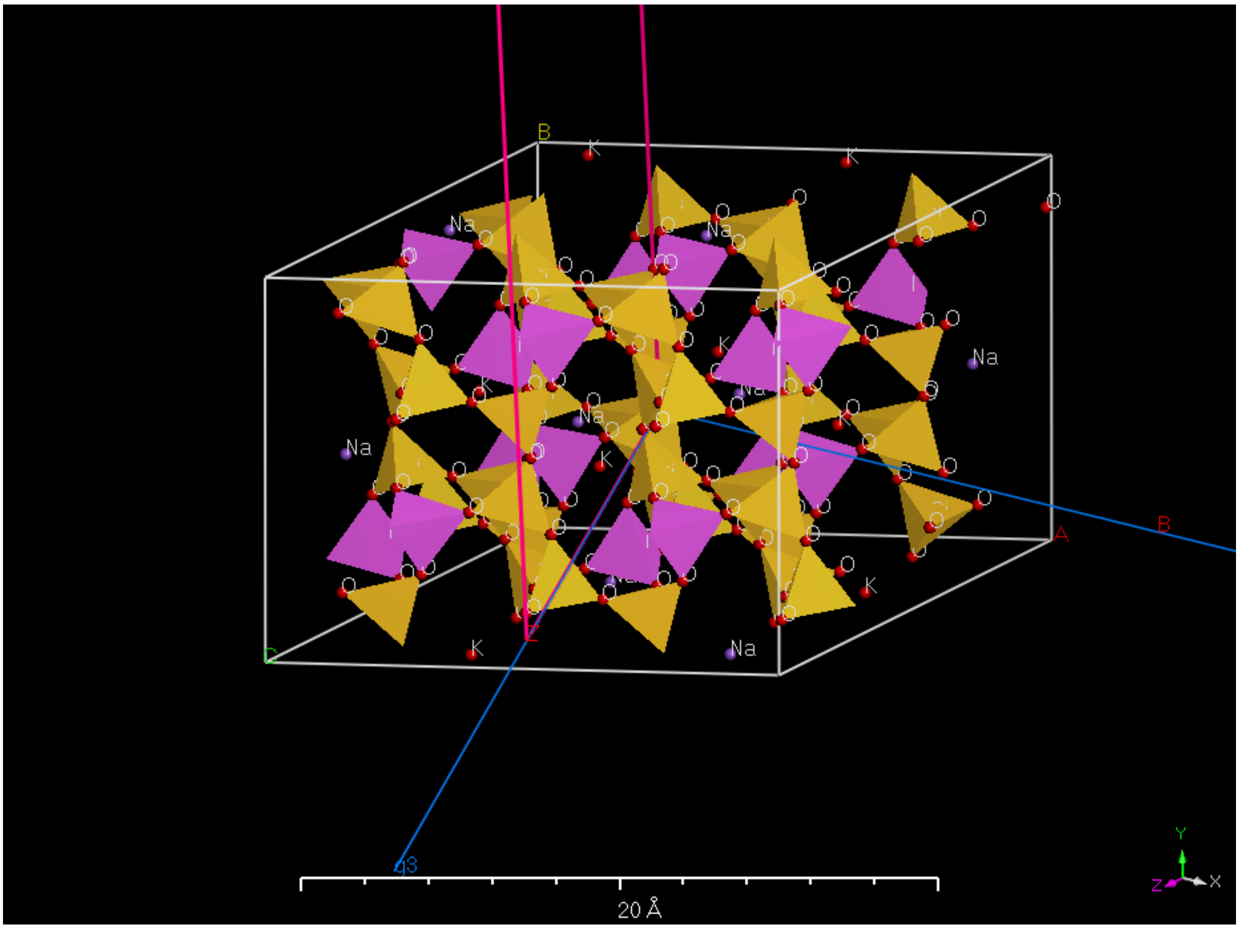

There are no symmetry operations specified or generated for this cell

Lattice parameters (A)		Cell Angles	
a =	16.390620	alpha =	92.233188
b =	12.755275	beta =	116.358277
c =	14.214170	gamma =	87.864763

Current cell volume = 2660.075315 A**3

BFGS: Final Enthalpy = -7.70262867E+004 eV

Element	Atom Number	Fractional coordinates of atoms u	v	w
O	1	0.003519	0.137991	0.491099
O	2	0.304668	1.001586	0.138725
O	3	0.408605	0.134138	0.105829
O	4	0.409232	0.855494	0.119640
O	5	0.005675	0.309971	0.128592
O	6	0.016025	0.698148	0.124929
O	7	0.099846	0.123396	0.200987
O	8	0.093674	0.875554	0.209565
O	9	0.255183	0.637078	0.491375
O	10	0.048865	0.501890	0.144721
O	11	0.158275	0.620539	0.102101
O	12	0.157554	0.358973	0.120497
O	13	0.257738	0.808487	0.128025

x	O	14	0.260498	0.196010	0.124865	x
x	O	15	0.349407	0.620505	0.198470	x
x	O	16	0.338952	0.372475	0.211907	x
x	O	17	0.500467	0.859442	0.010104	x
x	O	18	0.202218	0.000941	0.357034	x
x	O	19	0.094192	0.876106	0.396638	x
x	O	20	0.096821	0.146664	0.384287	x
x	O	21	0.491823	0.694858	0.369828	x
x	O	22	0.483994	0.303067	0.378416	x
x	O	23	0.398438	0.884556	0.300289	x
x	O	24	0.405324	0.126829	0.290716	x
x	O	25	0.246460	0.362812	0.009904	x
x	O	26	0.452214	0.499853	0.354808	x
x	O	27	0.341925	0.384819	0.399160	x
x	O	28	0.342115	0.641079	0.379043	x
x	O	29	0.246327	0.193930	0.370854	x
x	O	30	0.238519	0.803979	0.374754	x
x	O	31	0.150469	0.379567	0.300935	x
x	O	32	0.161314	0.626627	0.289752	x
x	O	33	0.501105	0.131359	0.490292	x
x	O	34	0.807715	1.001300	0.149757	x
x	O	35	0.908632	0.122261	0.099284	x
x	O	36	0.914336	0.857848	0.127473	x
x	O	37	0.510335	0.313201	0.125920	x
x	O	38	0.511112	0.692929	0.121916	x
x	O	39	0.598126	0.122231	0.197081	x
x	O	40	0.588767	0.868832	0.211934	x
x	O	41	0.745527	0.636520	0.488127	x
x	O	42	0.557679	0.502075	0.138034	x
x	O	43	0.661564	0.632337	0.105364	x
x	O	44	0.666029	0.357184	0.123842	x
x	O	45	0.761974	0.809487	0.131251	x
x	O	46	0.766796	0.194451	0.126397	x
x	O	47	0.852118	0.617204	0.200838	x
x	O	48	0.846634	0.371523	0.211114	x
x	O	49	0.993198	0.865884	0.007534	x
x	O	50	0.693222	0.000313	0.353686	x
x	O	51	0.589460	0.880077	0.399543	x
x	O	52	0.585563	0.143531	0.375135	x
x	O	53	0.992363	0.690729	0.374810	x
x	O	54	0.991063	0.307387	0.380330	x
x	O	55	0.903301	0.879393	0.305844	x
x	O	56	0.916616	0.131199	0.289699	x
x	O	57	0.754617	0.360743	0.011379	x
x	O	58	0.943915	0.500068	0.362581	x
x	O	59	0.839785	0.368587	0.394164	x
x	O	60	0.837294	0.646461	0.378715	x
x	O	61	0.738543	0.192044	0.371433	x
x	O	62	0.734726	0.806759	0.379060	x
x	O	63	0.649717	0.385762	0.300523	x
x	O	64	0.656778	0.629942	0.289141	x
x	O	65	-0.001382	0.139095	0.988498	x
x	O	66	0.299774	1.000443	0.645597	x
x	O	67	0.409812	0.116220	0.601977	x
x	O	68	0.412919	0.860743	0.625487	x
x	O	69	0.013614	0.306958	0.632523	x
x	O	70	0.010400	0.694121	0.621281	x
x	O	71	0.099996	0.110773	0.695044	x
x	O	72	0.091016	0.868865	0.711427	x
x	O	73	0.248071	0.635296	0.988924	x

x	O	74	0.055675	0.500554	0.639828	x
x	O	75	0.160817	0.629597	0.606277	x
x	O	76	0.165585	0.357031	0.626098	x
x	O	77	0.260814	0.807396	0.630799	x
x	O	78	0.265731	0.195535	0.622019	x
x	O	79	0.350836	0.613067	0.697686	x
x	O	80	0.345336	0.371672	0.709788	x
x	O	81	0.496868	0.865927	0.509911	x
x	O	82	0.193674	-0.002648	0.859149	x
x	O	83	0.086501	0.873094	0.894889	x
x	O	84	0.079932	0.137074	0.868816	x
x	O	85	0.489747	0.688410	0.871029	x
x	O	86	0.490281	0.305021	0.875341	x
x	O	87	0.401796	0.880354	0.803297	x
x	O	88	0.410878	0.128682	0.788283	x
x	O	89	0.251510	0.364674	0.510632	x
x	O	90	0.443576	0.497578	0.862049	x
x	O	91	0.340244	0.363815	0.893659	x
x	O	92	0.334833	0.642831	0.874138	x
x	O	93	0.234372	0.190588	0.869900	x
x	O	94	0.235612	0.804703	0.879109	x
x	O	95	0.151061	0.387570	0.803736	x
x	O	96	0.154322	0.629788	0.789557	x
x	O	97	0.501589	0.140148	0.989454	x
x	O	98	0.796978	1.001827	0.635484	x
x	O	99	0.910541	0.128176	0.608976	x
x	O	100	0.909305	0.858880	0.620001	x
x	O	101	0.508867	0.301992	0.631476	x
x	O	102	0.513531	0.697239	0.626005	x
x	O	103	0.602007	0.111939	0.698942	x
x	O	104	0.594534	0.873914	0.708896	x
x	O	105	0.755644	0.635261	0.992669	x
x	O	106	0.546418	0.499030	0.644606	x
x	O	107	0.656246	0.620543	0.603831	x
x	O	108	0.656469	0.359847	0.619402	x
x	O	109	0.755792	0.808374	0.625982	x
x	O	110	0.760268	0.198468	0.620306	x
x	O	111	0.847105	0.619960	0.696744	x
x	O	112	0.837041	0.375442	0.707735	x
x	O	113	1.004060	0.863471	0.513104	x
x	O	114	0.702518	-0.003481	0.861051	x
x	O	115	0.591905	0.869914	0.892984	x
x	O	116	0.591654	0.139832	0.878589	x
x	O	117	0.987863	0.693869	0.869015	x
x	O	118	0.983639	0.302738	0.869409	x
x	O	119	0.895745	0.885816	0.798080	x
x	O	120	0.899793	0.126176	0.789292	x
x	O	121	0.742814	0.366788	0.506398	x
x	O	122	0.951105	0.498726	0.853004	x
x	O	123	0.842629	0.377882	0.896387	x
x	O	124	0.838486	0.634638	0.876406	x
x	O	125	0.743419	0.190038	0.870344	x
x	O	126	0.738233	0.799864	0.873619	x
x	O	127	0.651330	0.378304	0.801189	x
x	O	128	0.657785	0.623617	0.790877	x
x	Na	1	0.366845	0.017935	0.425660	x
x	Na	2	0.885713	0.479111	0.074864	x
x	Na	3	0.616986	0.514224	0.424334	x
x	Na	4	0.136262	0.979788	0.581312	x
x	Na	5	0.381863	0.487004	0.573463	x

x	Na	6	0.115763	0.517309	0.923585	x
x	Na	7	0.632624	0.978994	0.573743	x
x	Na	8	0.864753	0.023080	0.925351	x
x	Al	1	0.003603	0.178242	0.106684	x
x	Al	2	0.252449	0.676399	0.106496	x
x	Al	3	0.493802	0.826391	0.392861	x
x	Al	4	0.248441	0.325804	0.393268	x
x	Al	5	0.503477	0.181723	0.105434	x
x	Al	6	0.758325	0.678095	0.108582	x
x	Al	7	1.000513	0.822603	0.395917	x
x	Al	8	0.743129	0.323603	0.391961	x
x	Al	9	0.005617	0.175749	0.608027	x
x	Al	10	0.256830	0.676106	0.607824	x
x	Al	11	0.497376	0.819941	0.892923	x
x	Al	12	0.243285	0.321696	0.893233	x
x	Al	13	0.505410	0.170305	0.608184	x
x	Al	14	0.749724	0.676125	0.605944	x
x	Al	15	0.989874	0.825411	0.890638	x
x	Al	16	0.748550	0.322087	0.892450	x
x	Si	1	0.003942	0.823797	0.116174	x
x	Si	2	0.348023	0.116903	0.164452	x
x	Si	3	0.343400	0.882909	0.175534	x
x	Si	4	0.250156	0.322375	0.115946	x
x	Si	5	0.098962	0.613614	0.164340	x
x	Si	6	0.092044	0.383301	0.177099	x
x	Si	7	0.495069	0.177097	0.384954	x
x	Si	8	0.154236	0.887468	0.335659	x
x	Si	9	0.160021	0.120258	0.324887	x
x	Si	10	0.250032	0.678010	0.384678	x
x	Si	11	0.401585	0.388541	0.336987	x
x	Si	12	0.407829	0.618302	0.322419	x
x	Si	13	0.501545	0.818870	0.114664	x
x	Si	14	0.853276	0.114094	0.165694	x
x	Si	15	0.847900	0.882631	0.181824	x
x	Si	16	0.757488	0.320160	0.117042	x
x	Si	17	0.600429	0.617023	0.163196	x
x	Si	18	0.597306	0.384746	0.175786	x
x	Si	19	1.003376	0.181046	0.387878	x
x	Si	20	0.648166	0.887003	0.336878	x
x	Si	21	0.652654	0.118762	0.321219	x
x	Si	22	0.744853	0.680723	0.384798	x
x	Si	23	0.902099	0.384288	0.337571	x
x	Si	24	0.905331	0.618493	0.325597	x
x	Si	25	0.001804	0.820566	0.615351	x
x	Si	26	0.349466	0.111999	0.663201	x
x	Si	27	0.345137	0.883045	0.679253	x
x	Si	28	0.255802	0.321274	0.615917	x
x	Si	29	0.098650	0.615844	0.663384	x
x	Si	30	0.097401	0.383531	0.678880	x
x	Si	31	0.499551	0.178850	0.883730	x
x	Si	32	0.148291	0.883611	0.837049	x
x	Si	33	0.151182	0.113762	0.819975	x
x	Si	34	0.244547	0.678641	0.883980	x
x	Si	35	0.401352	0.381988	0.836016	x
x	Si	36	0.403850	0.614941	0.822631	x
x	Si	37	0.503366	0.823316	0.616647	x
x	Si	38	0.844781	0.115677	0.662227	x
x	Si	39	0.840591	0.884095	0.673305	x
x	Si	40	0.748294	0.324476	0.612716	x
x	Si	41	0.596192	0.611547	0.665108	x

x	Si	42	0.591479	0.380589	0.677284	x
x	Si	43	0.991372	0.177333	0.880076	x
x	Si	44	0.654054	0.883042	0.835272	x
x	Si	45	0.658674	0.114177	0.823573	x
x	Si	46	0.748142	0.673998	0.884386	x
x	Si	47	0.900594	0.387283	0.832821	x
x	Si	48	0.905033	0.616254	0.820646	x
x	K	1	0.133911	0.993875	0.068468	x
x	K	2	0.386401	0.491322	0.068276	x
x	K	3	0.114605	0.508406	0.430606	x
x	K	4	0.636719	0.992279	0.069580	x
x	K	5	0.864756	0.008787	0.430809	x
x	K	6	0.364631	0.007262	0.932799	x
x	K	7	0.884579	0.493428	0.567486	x
x	K	8	0.615378	0.505612	0.933478	x

xxxxxxxxxxxxxxxxxxxxxxxxxxxxxxxxxxxxxxxxxxxxxxxxxxxxxxxxxxxxxxxxxxxxxxxxxxxxxx

Ab50Or50, cell 4 (supercell)

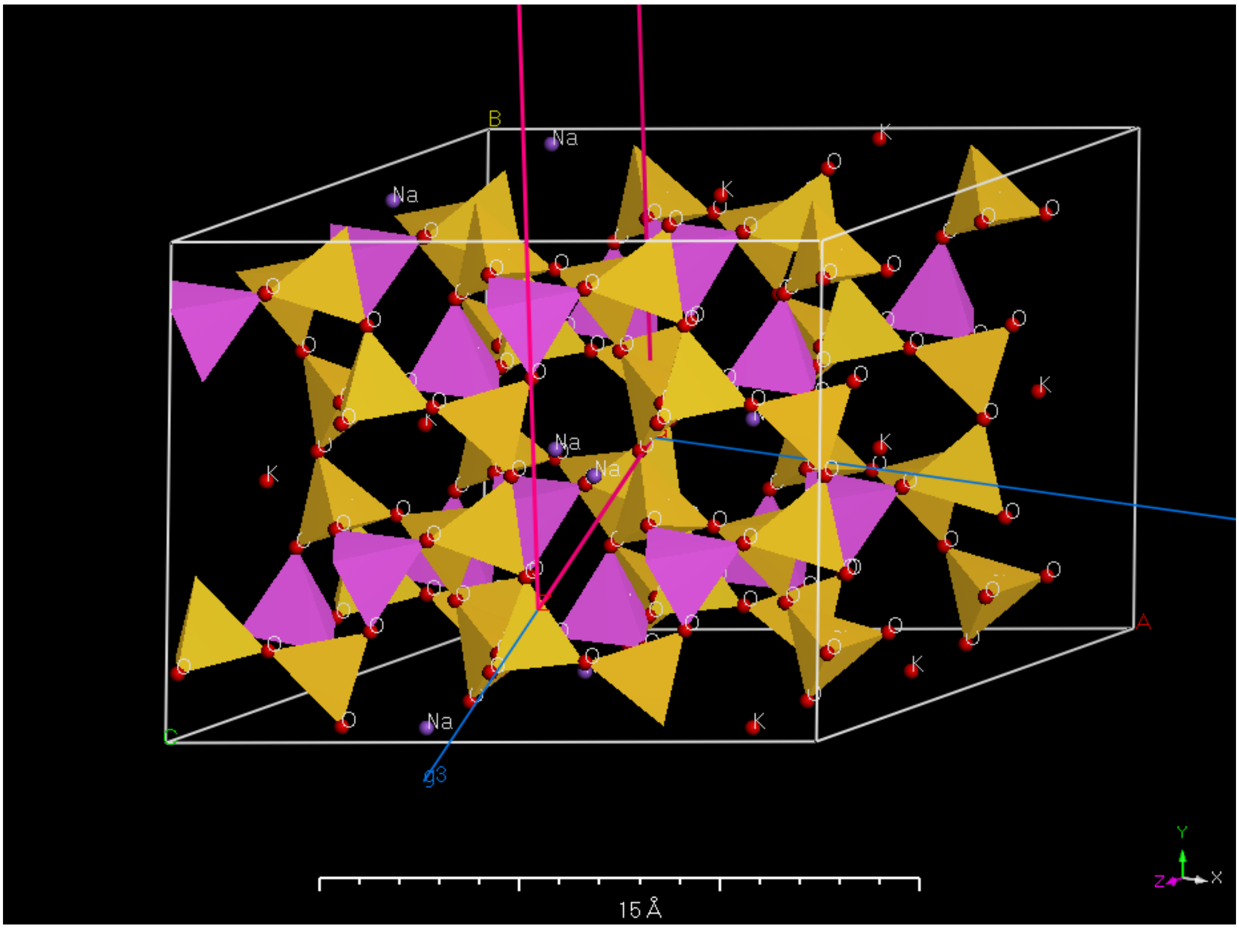

There are no symmetry operations specified or generated for this cell

Lattice parameters (A)		Cell Angles	
a =	16.378729	alpha =	92.576806
b =	12.735641	beta =	116.619905
c =	14.213852	gamma =	87.815985

Current cell volume = 2647.417439 A**3

BFGS: Final Enthalpy = -7.70266251E+004 eV

x	Element	Atom Number	Fractional coordinates of atoms u	v	w	x
x	O	1	0.012208	0.135271	0.491170	x
x	O	2	0.291840	1.000203	0.140233	x
x	O	3	0.399652	0.119066	0.097367	x
x	O	4	0.408898	0.860120	0.128275	x
x	O	5	0.017480	0.306422	0.130172	x
x	O	6	0.018792	0.695753	0.120734	x
x	O	7	0.108564	0.112590	0.197414	x
x	O	8	0.097230	0.872442	0.213578	x
x	O	9	0.260157	0.634347	0.491598	x
x	O	10	0.056575	0.501501	0.146573	x
x	O	11	0.166205	0.618837	0.106597	x
x	O	12	0.163408	0.367018	0.114939	x
x	O	13	0.258225	0.802955	0.131804	x

x	O	14	0.259512	0.198477	0.122736	x
x	O	15	0.356886	0.611313	0.196016	x
x	O	16	0.345077	0.372732	0.213992	x
x	O	17	0.489843	0.865653	0.008403	x
x	O	18	0.203432	0.000605	0.363474	x
x	O	19	0.095859	0.873618	0.398563	x
x	O	20	0.092421	0.153825	0.370672	x
x	O	21	0.491774	0.697046	0.368195	x
x	O	22	0.490489	0.301523	0.377263	x
x	O	23	0.393114	0.888688	0.303984	x
x	O	24	0.404924	0.127269	0.286007	x
x	O	25	0.258741	0.363703	0.012089	x
x	O	26	0.458160	0.499798	0.359766	x
x	O	27	0.350347	0.380934	0.402631	x
x	O	28	0.341102	0.639880	0.371725	x
x	O	29	0.249566	0.191682	0.370856	x
x	O	30	0.243821	0.805150	0.381324	x
x	O	31	0.160773	0.380333	0.300349	x
x	O	32	0.160935	0.632040	0.291026	x
x	O	33	0.491258	0.136297	0.487911	x
x	O	34	0.810206	1.000411	0.140935	x
x	O	35	0.916678	0.128023	0.105679	x
x	O	36	0.915214	0.854588	0.120264	x
x	O	37	0.500434	0.308318	0.129144	x
x	O	38	0.506179	0.694849	0.118677	x
x	O	39	0.589226	0.119666	0.199651	x
x	O	40	0.589065	0.867960	0.208974	x
x	O	41	0.743984	0.633672	0.487977	x
x	O	42	0.546567	0.499395	0.136526	x
x	O	43	0.654141	0.626382	0.101438	x
x	O	44	0.657579	0.346176	0.129328	x
x	O	45	0.760295	0.809862	0.125489	x
x	O	46	0.765927	0.192882	0.121195	x
x	O	47	0.845488	0.621153	0.200392	x
x	O	48	0.837361	0.371096	0.210015	x
x	O	49	1.006018	0.866328	0.012023	x
x	O	50	0.693426	-0.001502	0.353427	x
x	O	51	0.583795	0.881163	0.393403	x
x	O	52	0.586594	0.132982	0.385063	x
x	O	53	0.989705	0.690138	0.374511	x
x	O	54	0.984073	0.307118	0.378804	x
x	O	55	0.904513	0.878846	0.299607	x
x	O	56	0.912639	0.128904	0.289985	x
x	O	57	0.737792	0.364729	0.008829	x
x	O	58	0.939794	0.499589	0.359064	x
x	O	59	0.833321	0.371977	0.394321	x
x	O	60	0.834786	0.645412	0.379734	x
x	O	61	0.732520	0.193577	0.369827	x
x	O	62	0.731208	0.804247	0.379266	x
x	O	63	0.641436	0.387409	0.302586	x
x	O	64	0.652771	0.627558	0.286422	x
x	O	65	0.012208	0.135270	0.991170	x
x	O	66	0.291839	1.000202	0.640234	x
x	O	67	0.399652	0.119066	0.597367	x
x	O	68	0.408899	0.860120	0.628276	x
x	O	69	0.017480	0.306423	0.630173	x
x	O	70	0.018792	0.695753	0.620733	x
x	O	71	0.108564	0.112591	0.697414	x
x	O	72	0.097230	0.872443	0.713577	x
x	O	73	0.260157	0.634348	0.991598	x

x	O	74	0.056574	0.501502	0.646572	x
x	O	75	0.166205	0.618837	0.606598	x
x	O	76	0.163407	0.367018	0.614937	x
x	O	77	0.258226	0.802954	0.631805	x
x	O	78	0.259511	0.198477	0.622737	x
x	O	79	0.356886	0.611312	0.696016	x
x	O	80	0.345076	0.372731	0.713993	x
x	O	81	0.489843	0.865653	0.508403	x
x	O	82	0.203433	0.000605	0.863474	x
x	O	83	0.095858	0.873619	0.898561	x
x	O	84	0.092421	0.153824	0.870672	x
x	O	85	0.491774	0.697045	0.868196	x
x	O	86	0.490489	0.301522	0.877264	x
x	O	87	0.393114	0.888688	0.803984	x
x	O	88	0.404924	0.127268	0.786007	x
x	O	89	0.258743	0.363702	0.512089	x
x	O	90	0.458161	0.499798	0.859767	x
x	O	91	0.350348	0.380934	0.902633	x
x	O	92	0.341101	0.639879	0.871724	x
x	O	93	0.249566	0.191683	0.870856	x
x	O	94	0.243820	0.805151	0.881322	x
x	O	95	0.160774	0.380335	0.800349	x
x	O	96	0.160934	0.632040	0.791026	x
x	O	97	0.491259	0.136297	0.987911	x
x	O	98	0.810206	1.000411	0.640935	x
x	O	99	0.916679	0.128023	0.605679	x
x	O	100	0.915214	0.854588	0.620266	x
x	O	101	0.500434	0.308318	0.629144	x
x	O	102	0.506180	0.694849	0.618677	x
x	O	103	0.589226	0.119666	0.699651	x
x	O	104	0.589065	0.867959	0.708974	x
x	O	105	0.743983	0.633671	0.987977	x
x	O	106	0.546568	0.499395	0.636526	x
x	O	107	0.654141	0.626381	0.601436	x
x	O	108	0.657579	0.346176	0.629328	x
x	O	109	0.760294	0.809862	0.625489	x
x	O	110	0.765927	0.192882	0.621195	x
x	O	111	0.845487	0.621153	0.700393	x
x	O	112	0.837362	0.371096	0.710016	x
x	O	113	1.006015	0.866329	0.512022	x
x	O	114	0.693425	-0.001502	0.853427	x
x	O	115	0.583795	0.881163	0.893403	x
x	O	116	0.586593	0.132981	0.885063	x
x	O	117	0.989705	0.690138	0.874510	x
x	O	118	0.984074	0.307118	0.878805	x
x	O	119	0.904512	0.878847	0.799608	x
x	O	120	0.912639	0.128904	0.789985	x
x	O	121	0.737793	0.364730	0.508830	x
x	O	122	0.939794	0.499589	0.859066	x
x	O	123	0.833322	0.371977	0.894321	x
x	O	124	0.834787	0.645412	0.879736	x
x	O	125	0.732520	0.193577	0.869827	x
x	O	126	0.731208	0.804246	0.879268	x
x	O	127	0.641437	0.387409	0.802586	x
x	O	128	0.652771	0.627557	0.786422	x
x	Na	1	0.132452	0.985688	0.074101	x
x	Na	2	0.384316	0.487702	0.070231	x
x	Na	3	0.365681	0.012303	0.429768	x
x	Na	4	0.617549	0.514316	0.425902	x
x	Na	5	0.132452	0.985686	0.574102	x

x	Na	6	0.384318	0.487698	0.570233	x
x	Na	7	0.365683	0.012300	0.929770	x
x	Na	8	0.617549	0.514313	0.925902	x
x	Al	1	0.013242	0.174666	0.107868	x
x	Al	2	0.260074	0.671670	0.107829	x
x	Al	3	0.489925	0.828332	0.392171	x
x	Al	4	0.255684	0.323528	0.394948	x
x	Al	5	0.494316	0.176471	0.105052	x
x	Al	6	0.749962	0.677695	0.105900	x
x	Al	7	1.000038	0.822304	0.394099	x
x	Al	8	0.736758	0.325333	0.392132	x
x	Al	9	0.013242	0.174667	0.607868	x
x	Al	10	0.260074	0.671668	0.607829	x
x	Al	11	0.489926	0.828331	0.892171	x
x	Al	12	0.255684	0.323529	0.894948	x
x	Al	13	0.494316	0.176471	0.605052	x
x	Al	14	0.749962	0.677696	0.605900	x
x	Al	15	1.000038	0.822304	0.894099	x
x	Al	16	0.736758	0.325333	0.892132	x
x	Si	1	0.008755	0.821903	0.116245	x
x	Si	2	0.341659	0.112512	0.160819	x
x	Si	3	0.338922	0.884092	0.178912	x
x	Si	4	0.256742	0.324574	0.115410	x
x	Si	5	0.104289	0.614156	0.165193	x
x	Si	6	0.101053	0.383860	0.176507	x
x	Si	7	0.493258	0.175426	0.384590	x
x	Si	8	0.157305	0.885868	0.339992	x
x	Si	9	0.162755	0.118358	0.322806	x
x	Si	10	0.253071	0.678470	0.385481	x
x	Si	11	0.408341	0.387488	0.339180	x
x	Si	12	0.411078	0.615909	0.321088	x
x	Si	13	0.496929	0.821529	0.114519	x
x	Si	14	0.854758	0.115258	0.163503	x
x	Si	15	0.848484	0.881056	0.174897	x
x	Si	16	0.747831	0.318617	0.115777	x
x	Si	17	0.592696	0.614132	0.160009	x
x	Si	18	0.587244	0.381642	0.177194	x
x	Si	19	1.002168	0.181383	0.384223	x
x	Si	20	0.645710	0.885844	0.334807	x
x	Si	21	0.648947	0.116140	0.323493	x
x	Si	22	0.741246	0.678097	0.383755	x
x	Si	23	0.895242	0.384742	0.336496	x
x	Si	24	0.901517	0.618944	0.325102	x
x	Si	25	0.008754	0.821904	0.616245	x
x	Si	26	0.341659	0.112512	0.660820	x
x	Si	27	0.338922	0.884092	0.678912	x
x	Si	28	0.256742	0.324574	0.615410	x
x	Si	29	0.104289	0.614157	0.665193	x
x	Si	30	0.101053	0.383861	0.676507	x
x	Si	31	0.493258	0.175426	0.884590	x
x	Si	32	0.157304	0.885868	0.839990	x
x	Si	33	0.162756	0.118358	0.822806	x
x	Si	34	0.253070	0.678471	0.885481	x
x	Si	35	0.408341	0.387488	0.839181	x
x	Si	36	0.411078	0.615908	0.821088	x
x	Si	37	0.496930	0.821529	0.614519	x
x	Si	38	0.854758	0.115258	0.663503	x
x	Si	39	0.848483	0.881056	0.674898	x
x	Si	40	0.747832	0.318617	0.615777	x
x	Si	41	0.592696	0.614132	0.660009	x

x	Si	42	0.587245	0.381642	0.677194	x
x	Si	43	1.002169	0.181383	0.884223	x
x	Si	44	0.645711	0.885843	0.834808	x
x	Si	45	0.648947	0.116139	0.823493	x
x	Si	46	0.741246	0.678096	0.883755	x
x	Si	47	0.895243	0.384742	0.836497	x
x	Si	48	0.901516	0.618944	0.825103	x
x	K	1	0.113950	0.505229	0.426368	x
x	K	2	0.636051	0.994769	0.073634	x
x	K	3	0.884708	0.489434	0.073784	x
x	K	4	0.865293	0.010566	0.426216	x
x	K	5	0.113950	0.505230	0.926367	x
x	K	6	0.636050	0.994771	0.573632	x
x	K	7	0.884707	0.489434	0.573783	x
x	K	8	0.865292	0.010566	0.926215	x

xxxxxxxxxxxxxxxxxxxxxxxxxxxxxxxxxxxxxxxxxxxxxxxxxxxxxxxxxxxxxxxxxxxxxxxxxxxxxxx

Ab25Or75

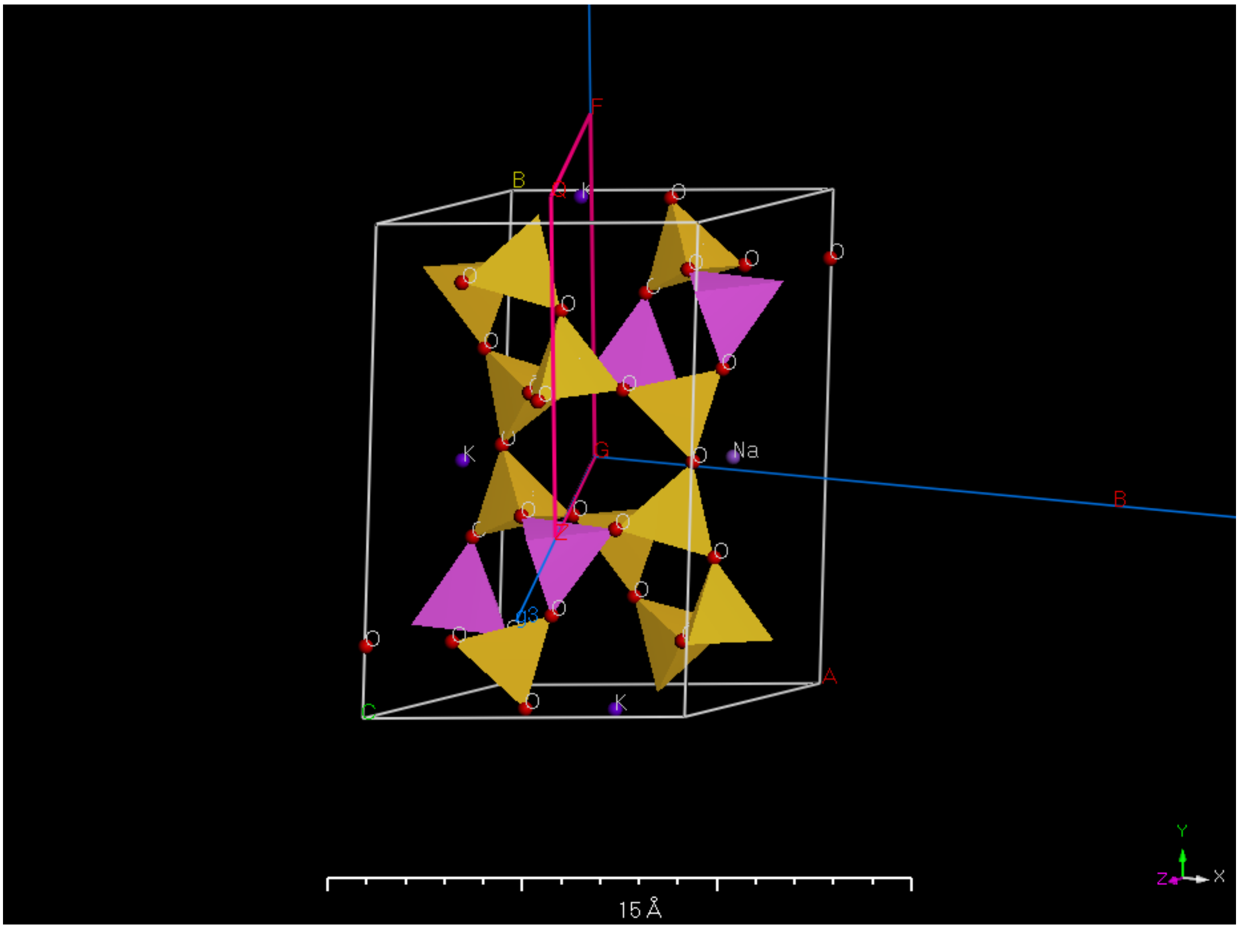

There are no symmetry operations specified or generated for this cell

	Lattice parameters(A)		Cell Angles
a =	8.282419	alpha =	91.631586
b =	12.801427	beta =	115.982137
c =	7.116972	gamma =	87.773390

Current cell volume = 677.756569 A**3

BFGS: Final Enthalpy = -1.88869974E+004 eV

Element	Atom Number	Fractional coordinates of atoms u	v	w
O	1	-0.002374	0.144800	0.988387
O	2	0.615400	1.001811	0.286329
O	3	0.816916	0.137376	0.219879
O	4	0.829275	0.862452	0.238017
O	5	0.016871	0.316828	0.263150
O	6	0.032868	0.697680	0.250196
O	7	0.192276	0.126230	0.403912
O	8	0.185208	0.874609	0.409623
O	9	0.508347	0.638563	0.978519
O	10	0.110641	0.504466	0.287313
O	11	0.328423	0.629924	0.217228
O	12	0.315998	0.358100	0.235355
O	13	0.530054	0.808119	0.251854

x	O	14	0.524414	0.195077	0.261612	x
x	O	15	0.704798	0.613890	0.389021	x
x	O	16	0.673579	0.372943	0.421954	x
x	O	17	0.999875	0.860033	0.010499	x
x	O	18	0.382292	-0.001345	0.710243	x
x	O	19	0.178859	0.865731	0.778432	x
x	O	20	0.171774	0.138205	0.764437	x
x	O	21	0.978743	0.686234	0.737410	x
x	O	22	0.968819	0.303948	0.739848	x
x	O	23	0.805006	0.877697	0.596432	x
x	O	24	0.814516	0.126133	0.589609	x
x	O	25	0.502521	0.360394	0.024470	x
x	O	26	0.884573	0.496728	0.713930	x
x	O	27	0.685614	0.366678	0.800375	x
x	O	28	0.670275	0.644756	0.737581	x
x	O	29	0.473398	0.189280	0.751451	x
x	O	30	0.472570	0.805751	0.741804	x
x	O	31	0.309095	0.380912	0.601394	x
x	O	32	0.316420	0.629377	0.582219	x
x	Na	1	0.779110	0.469100	0.164985	x
x	Al	1	0.004606	0.185503	0.220860	x
x	Al	2	0.516368	0.676723	0.212869	x
x	Al	3	0.991758	0.817342	0.778501	x
x	Al	4	0.492646	0.320258	0.789750	x
x	Si	1	0.010043	0.822511	0.225621	x
x	Si	2	0.699155	0.116766	0.338422	x
x	Si	3	0.696605	0.882640	0.349703	x
x	Si	4	0.501995	0.320427	0.233488	x
x	Si	5	0.203715	0.617388	0.331586	x
x	Si	6	0.191740	0.385907	0.355193	x
x	Si	7	0.989629	0.179146	0.772447	x
x	Si	8	0.298293	0.883774	0.661036	x
x	Si	9	0.302693	0.117804	0.650200	x
x	Si	10	0.494667	0.679960	0.764218	x
x	Si	11	0.796802	0.383439	0.671554	x
x	Si	12	0.807537	0.614915	0.637802	x
x	K	1	0.270473	0.995133	0.131968	x
x	K	2	0.726837	0.006540	0.867283	x
x	K	3	0.228283	0.511343	0.860029	x

xxxxxxxxxxxxxxxxxxxxxxxxxxxxxxxxxxxxxxxxxxxxxxxxxxxxxxxxxxxxxxxxxxxxxxxx

Or100, Low microcline

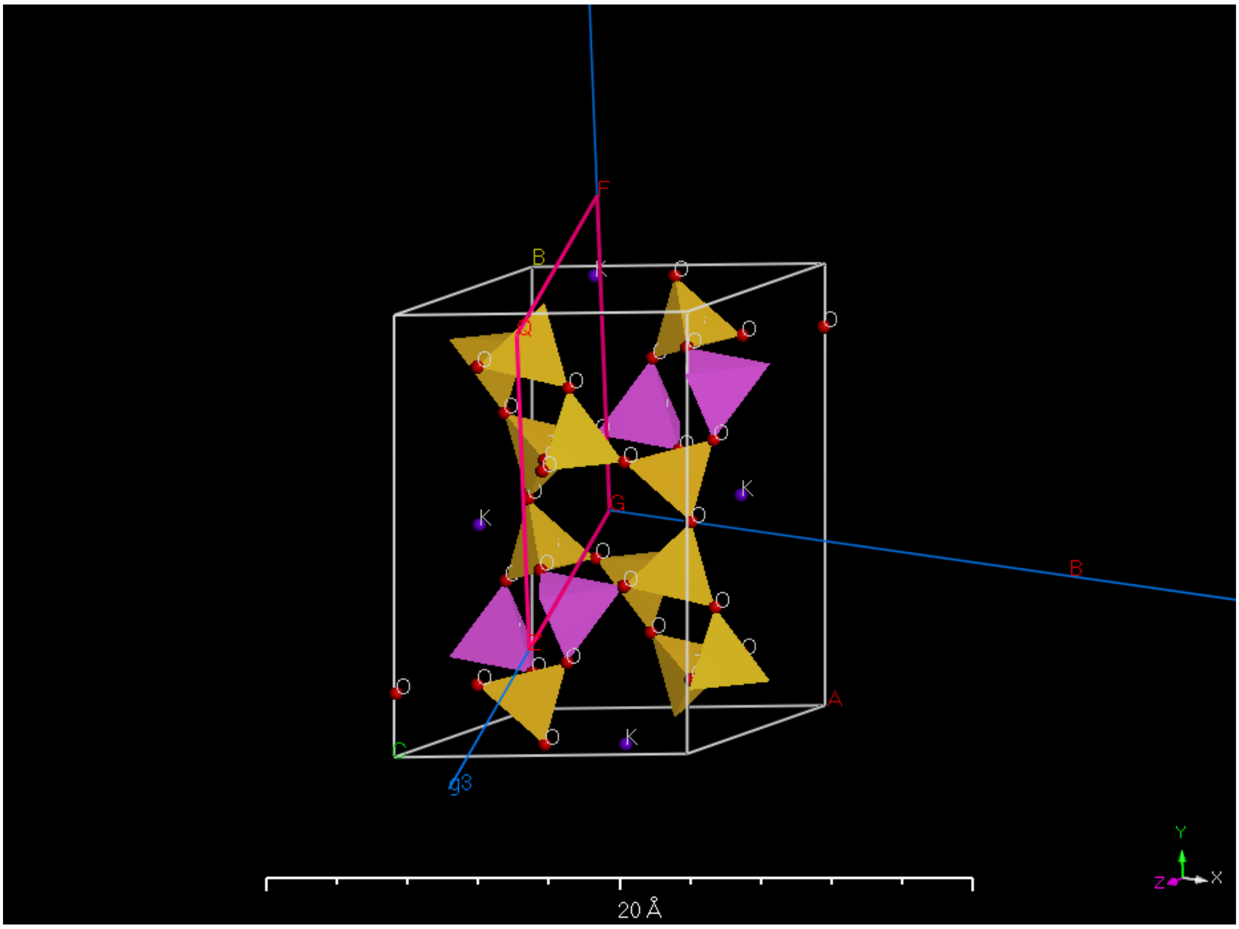


```
Number of symmetry operations   =          4
Point group of crystal =     2: Ci, -1, -1

     Lattice parameters(A)       Cell Angles
 a =     8.368703          alpha =   90.575916
 b =    12.821519          beta  =  116.068261
 c =     7.122902          gamma =   87.707884

Current cell volume =  685.960642 A**3

BFGS: Final Enthalpy     = -1.85175282E+004 eV
```

x	Element	Atom Number	Fractional coordinates of atoms u	v	w	x
x	O	1	-0.000050	0.142413	0.987862	x
x	O	2	0.621312	1.005512	0.285715	x
x	O	3	0.820102	0.142867	0.219996	x
x	O	4	0.826374	0.863437	0.233680	x
x	O	5	0.027457	0.316582	0.248750	x
x	O	6	0.031398	0.697776	0.267726	x
x	O	7	0.191762	0.123899	0.404907	x
x	O	8	0.179389	0.875934	0.409648	x
x	O	9	0.499950	0.642413	0.987862	x
x	O	10	1.121312	1.505512	0.285715	x
x	O	11	1.320102	0.642867	0.219996	x
x	O	12	1.326374	1.363437	0.233680	x
x	O	13	0.527457	0.816582	0.248750	x

x	O	14	0.531398	1.197776	0.267726	x
x	O	15	0.691762	0.623899	0.404907	x
x	O	16	0.679389	1.375934	0.409648	x
x	O	17	0.000050	-0.142413	-0.987862	x
x	O	18	-0.621312	-1.005512	-0.285715	x
x	O	19	-0.820102	-0.142867	-0.219996	x
x	O	20	-0.826374	-0.863437	-0.233680	x
x	O	21	-0.027457	-0.316582	-0.248750	x
x	O	22	-0.031398	-0.697776	-0.267726	x
x	O	23	-0.191762	-0.123899	-0.404907	x
x	O	24	-0.179389	-0.875934	-0.409648	x
x	O	25	0.500050	0.357587	-0.987862	x
x	O	26	-0.121312	-0.505512	-0.285715	x
x	O	27	-0.320102	0.357133	-0.219996	x
x	O	28	-0.326374	-0.363437	-0.233680	x
x	O	29	0.472543	0.183418	-0.248750	x
x	O	30	0.468602	-0.197776	-0.267726	x
x	O	31	0.308238	0.376101	-0.404907	x
x	O	32	0.320611	-0.375934	-0.409648	x
x	Al	1	0.008297	0.185655	0.218101	x
x	Al	2	0.508297	0.685655	0.218101	x
x	Al	3	-0.008297	-0.185655	-0.218101	x
x	Al	4	0.491703	0.314345	-0.218101	x
x	Si	1	0.007848	0.822421	0.228592	x
x	Si	2	0.704771	0.119436	0.339075	x
x	Si	3	0.698388	0.885489	0.348764	x
x	Si	4	0.507848	1.322421	0.228592	x
x	Si	5	1.204771	0.619436	0.339075	x
x	Si	6	1.198388	1.385489	0.348764	x
x	Si	7	-0.007848	-0.822421	-0.228592	x
x	Si	8	-0.704771	-0.119436	-0.339075	x
x	Si	9	-0.698388	-0.885489	-0.348764	x
x	Si	10	0.492152	-0.322421	-0.228592	x
x	Si	11	-0.204771	0.380564	-0.339075	x
x	Si	12	-0.198388	-0.385489	-0.348764	x
x	K	1	0.274799	0.991466	0.134659	x
x	K	2	0.774799	1.491466	0.134659	x
x	K	3	-0.274799	-0.991466	-0.134659	x
x	K	4	0.225201	-0.491466	-0.134659	x

xxxxxxxxxxxxxxxxxxxxxxxxxxxxxxxxxxxxxxxxxxxxxxxxxxxxxxxxxxxxxxxxxxxxxxx

Excess volumes of mixing (V^{exc}) plotted against composition (X_{Or}):

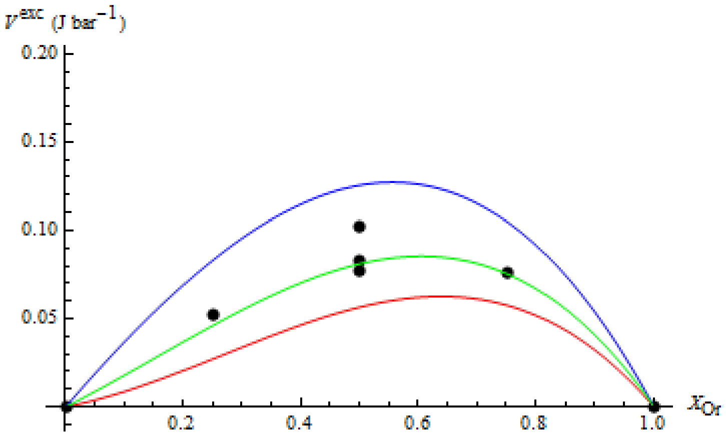

Black symbols: DFT data from this study.
Curves: Margules fit through excess volumes derived from X-ray diffraction studies of Kroll et al. 1986 (*red*), Hovis and Peckins 1978 (*green*), and Hovis 1988 (*blue*).
