# Supplementary material for: First-principles investigation of the lattice vibrations in the alkali feldspar solid solution
Source: Phys Chem Miner. 2014 Oct 11;42(3):243–9. doi: 10.1007/s00269-014-0715-8 (PMC4509561; doi:10.1007/s00269-014-0715-8)

Appendix B:

Phonon dispersion relations in the low frequency range of the end-members and all investigated $Ab_{50}Or_{50}$ cells of the study "First-principles investigation of the lattice vibrations in the alkali feldspar solid solution" published by Artur Benisek, Edgar Dachs, Michael Grodzicki in Physics and Chemistry of Minerals.

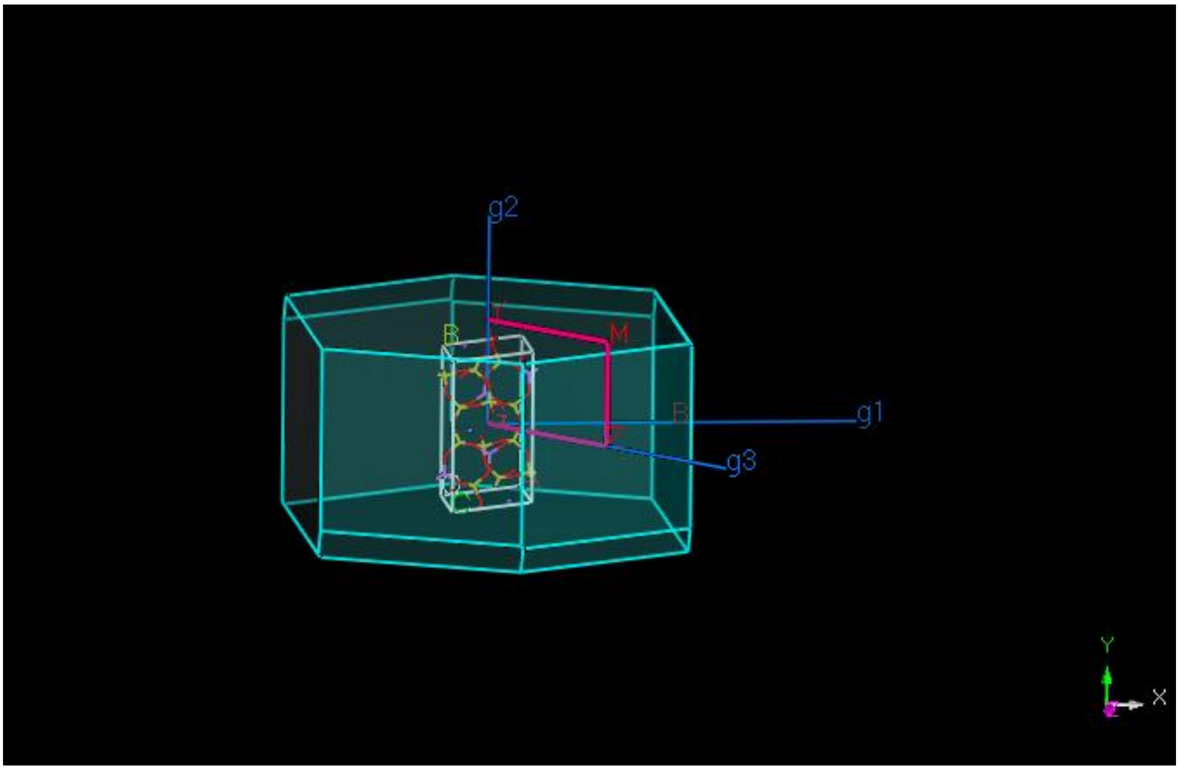

Brillouin zone of feldspar unit cell. Path of phonon dispersion relation #1: Γ-Y-M-Z-Γ.

Symmetry points	Coordinates
Γ	0, 0, 0
Y	0, ½, 0
M	0, ½, ½
Z	0, 0, ½
Γ	0, 0, 0

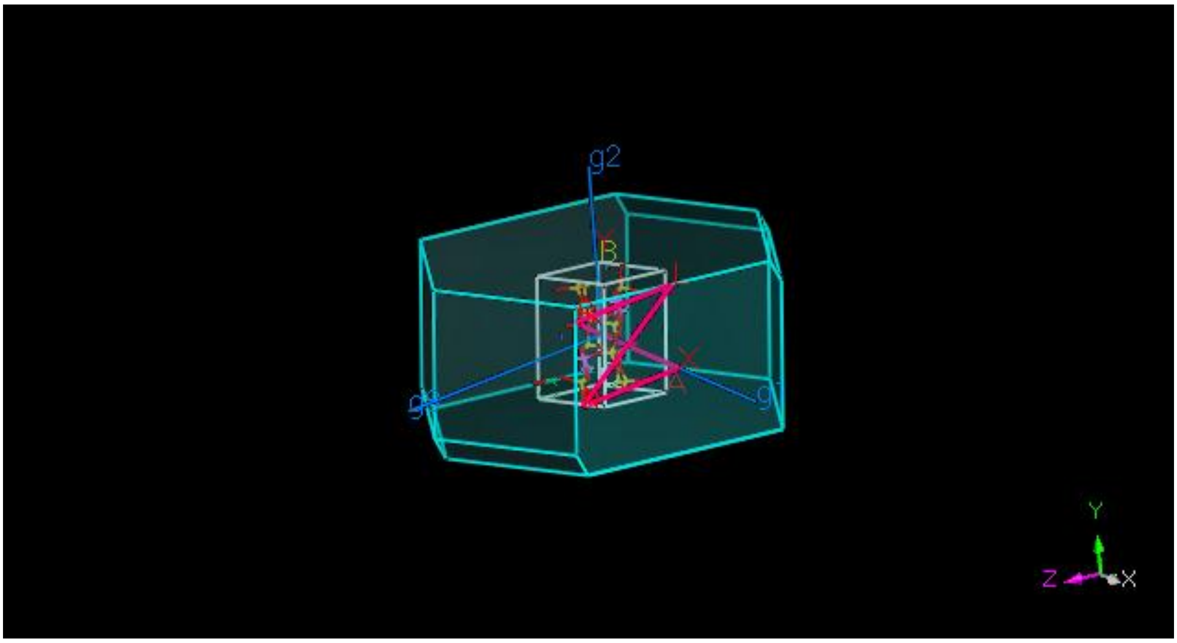

Brillouin zone of feldspar unit cell. Path of phonon dispersion relation #2: Γ-X-N-L-R-Γ.

Symmetry points	Coordinates
Γ	0, 0, 0
X	½, 0, 0
N	½, 0, ½
L	½, ½, 0
R	½, ½, ½
Γ	0, 0, 0

Low albite

Phonon dispersion relation #1

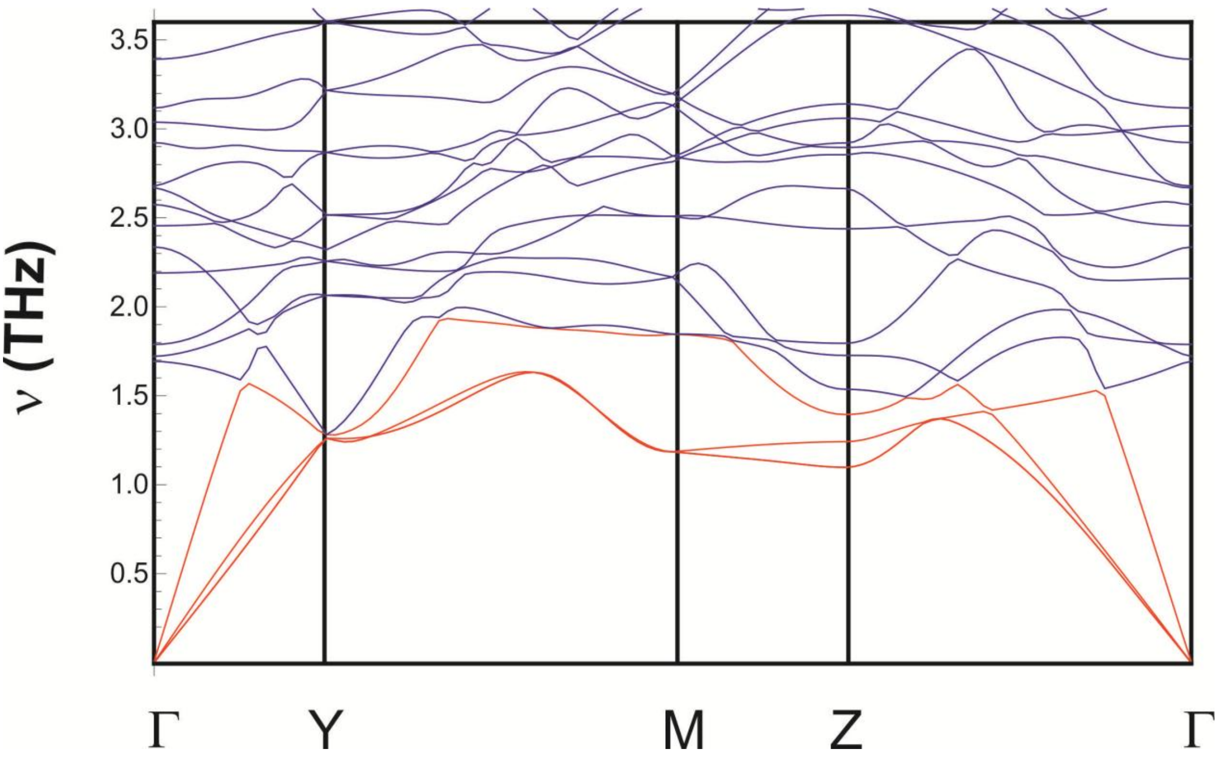

Phonon dispersion relation #2

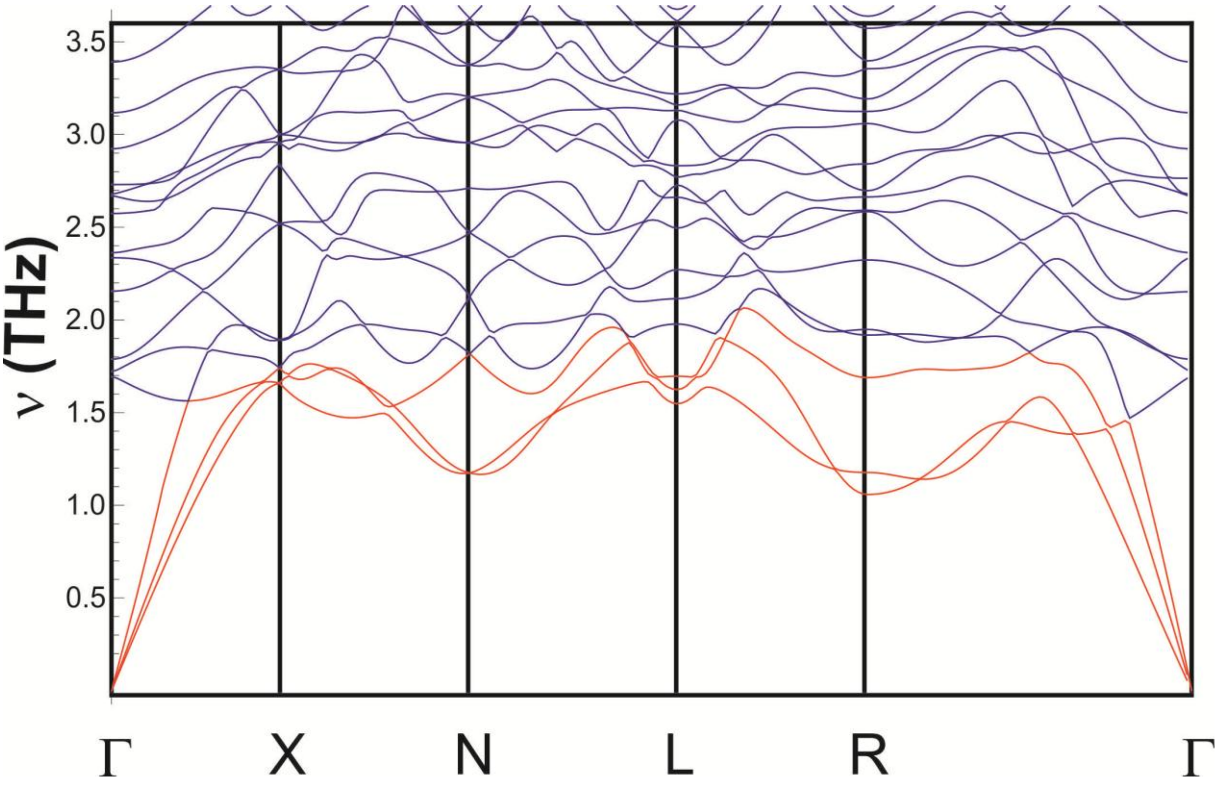

Low microcline

Phonon dispersion relation #1

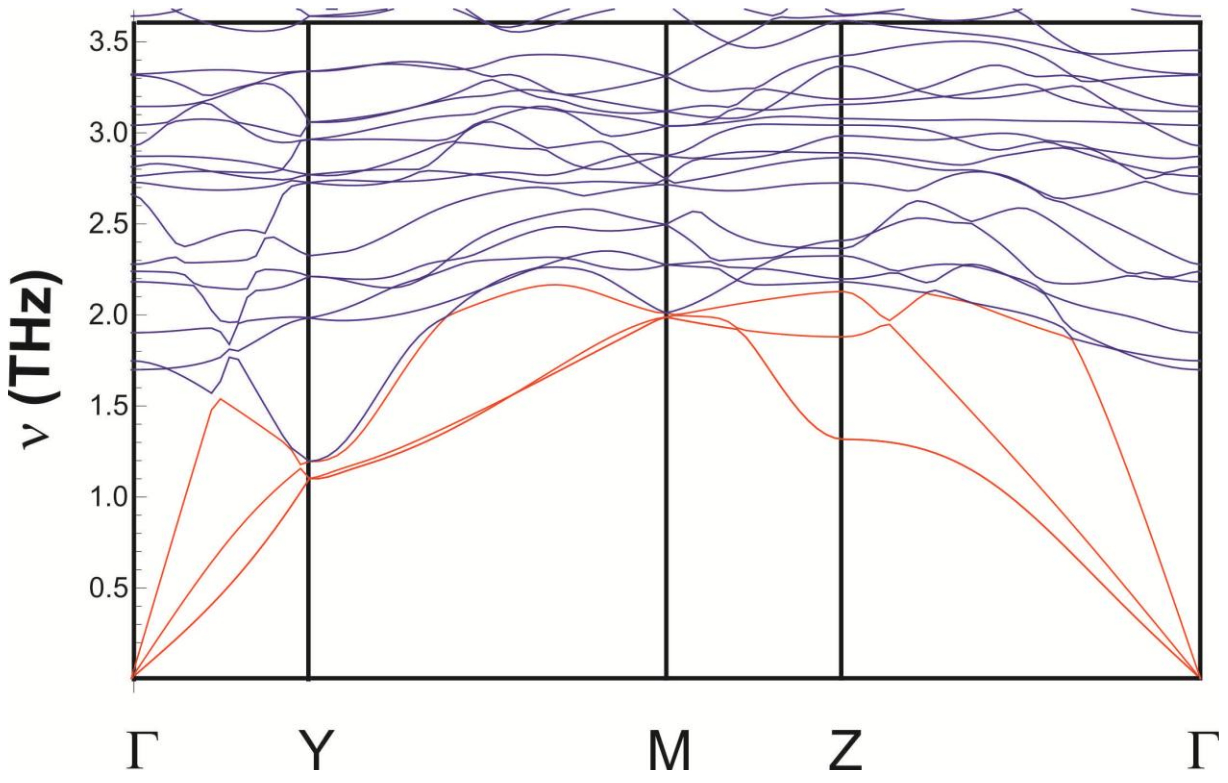

Phonon dispersion relation #2

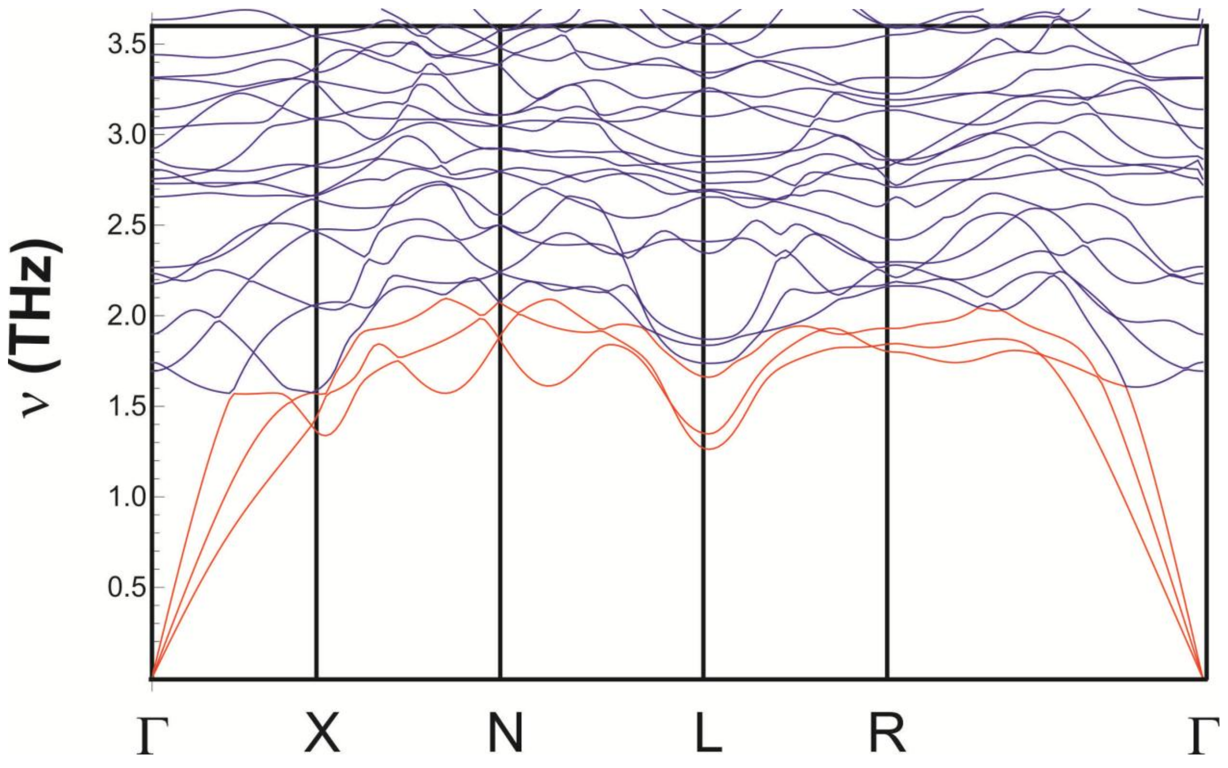

Ab$_{50}$Or$_{50}$, cell 1

Phonon dispersion relation #1

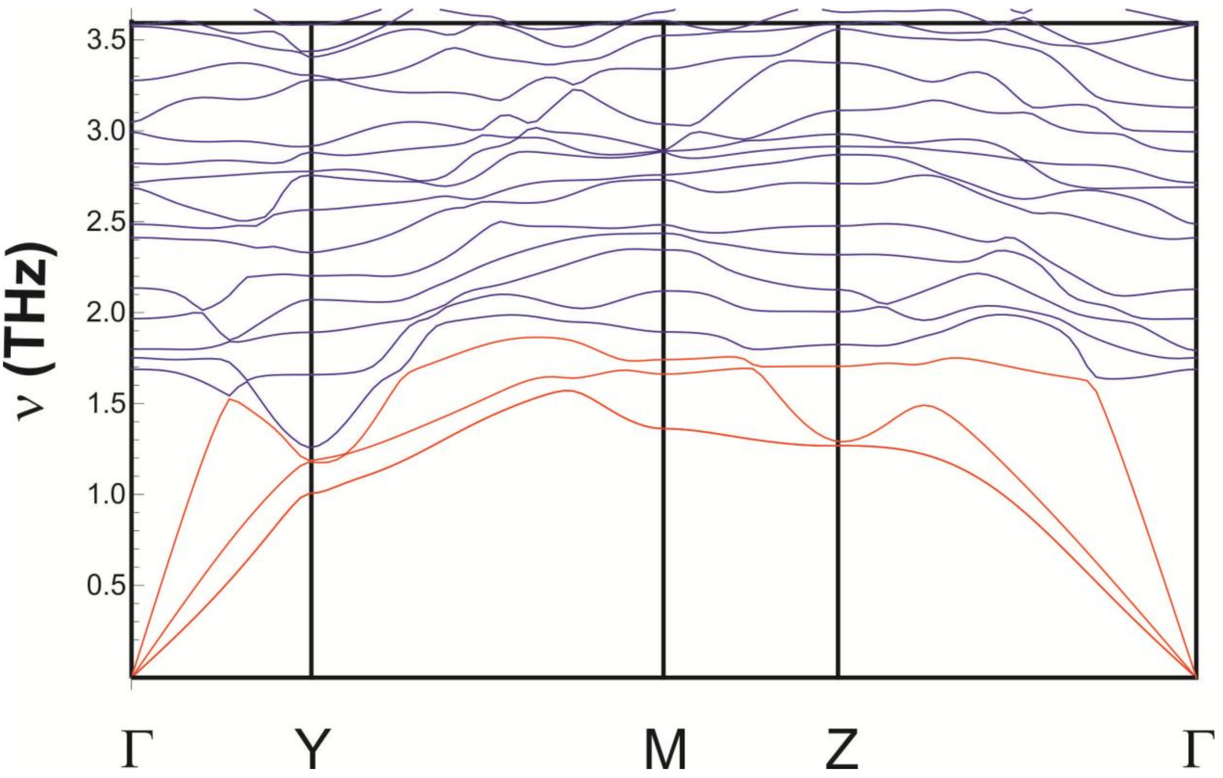

Phonon dispersion relation #2

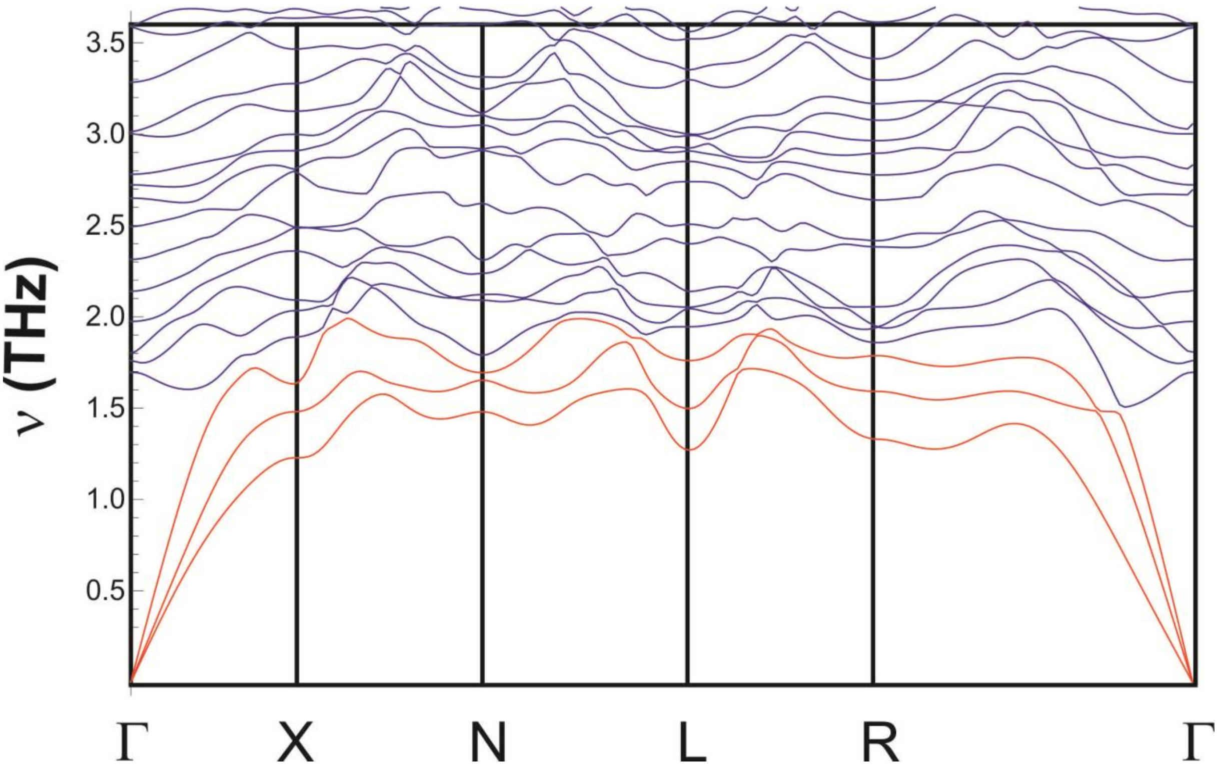

$Ab_{50}Or_{50}$, cell 2

Phonon dispersion relation #1

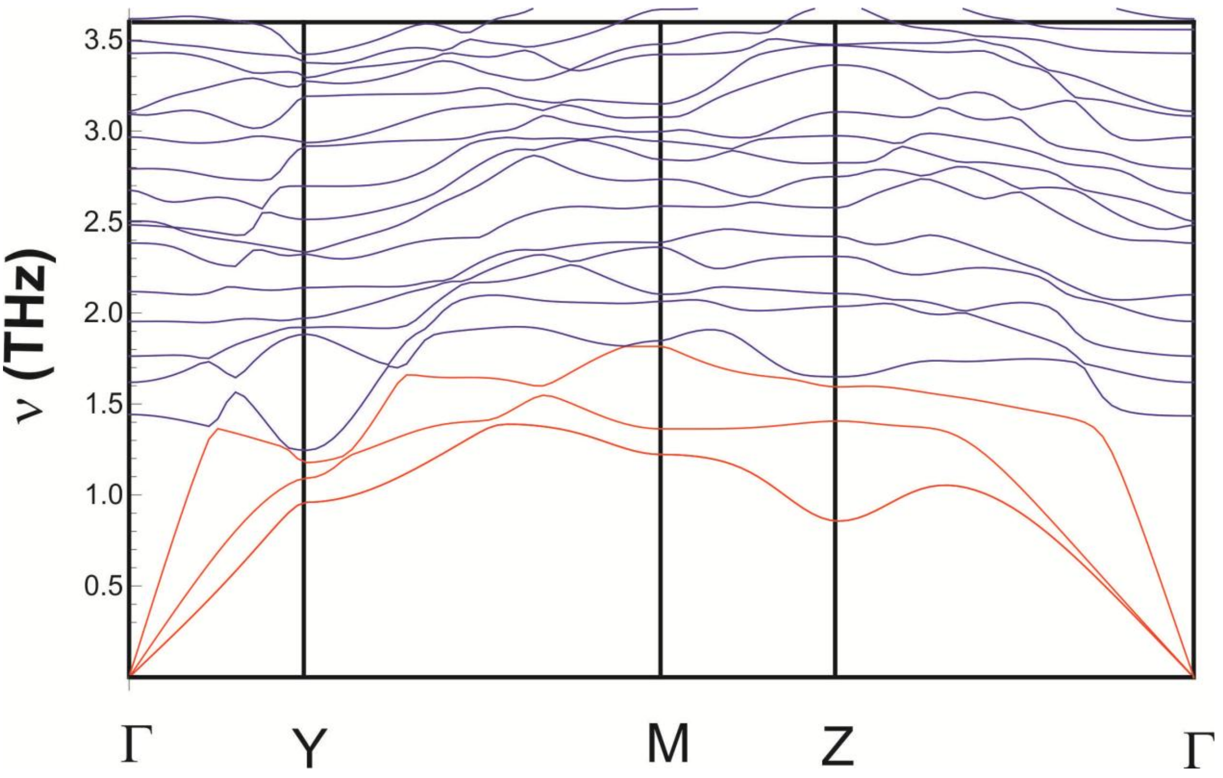

Phonon dispersion relation #2

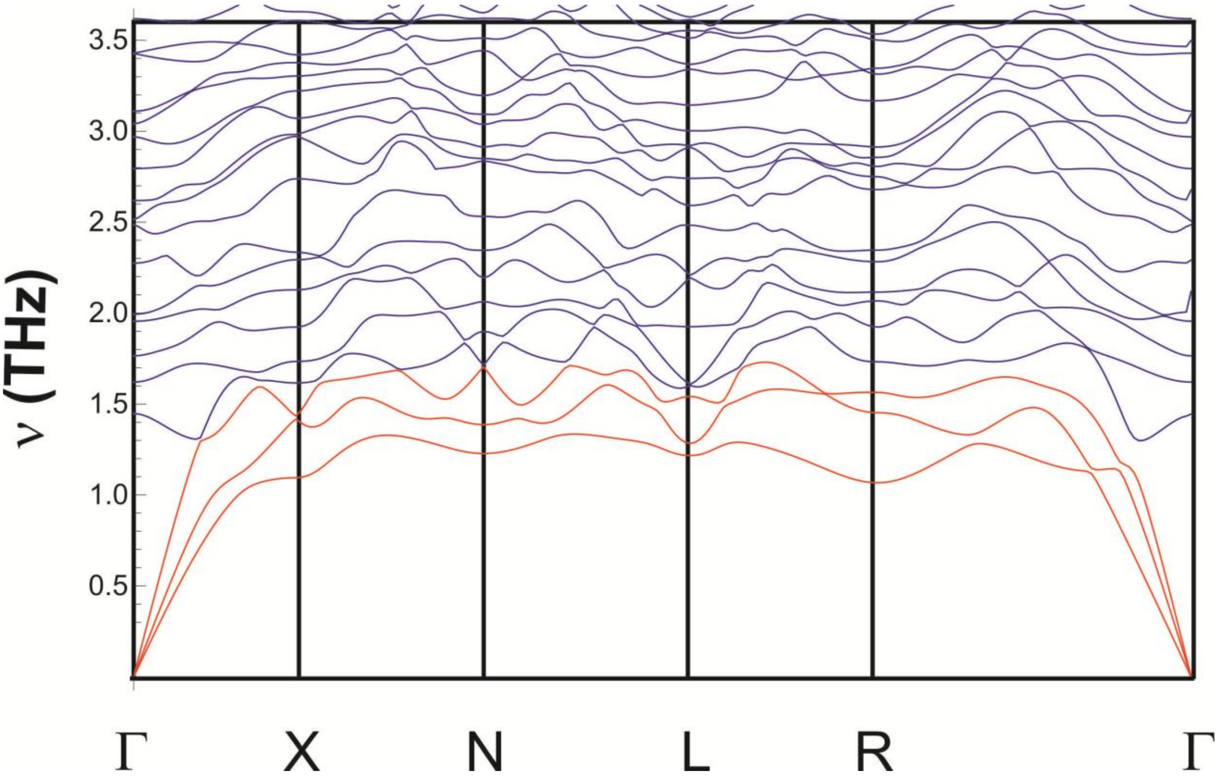

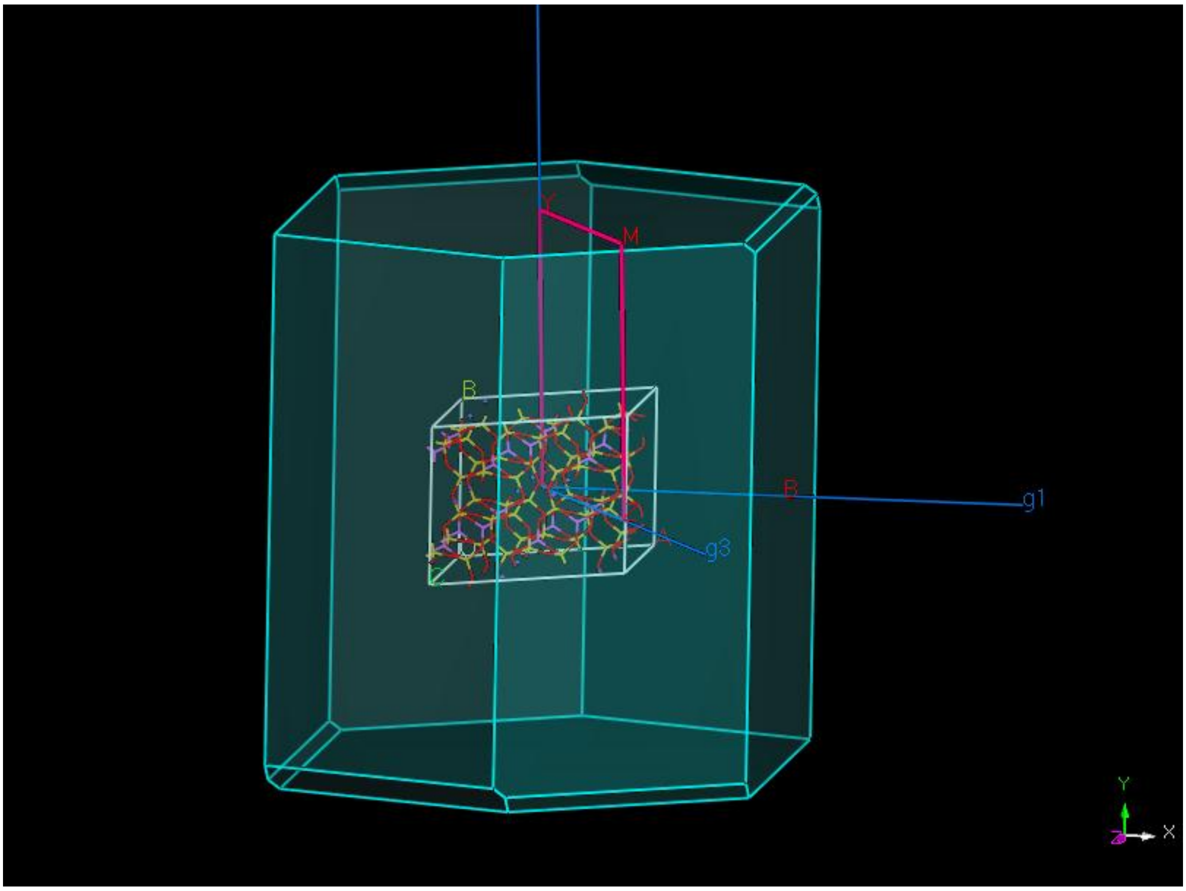

Brillouin zone of the supercells (cell 3 and 4 with $Ab_{50}Or_{50}$ composition).
Path: Γ-Y-M-Z-Γ.

Symmetry points	Coordinates
Γ	0, 0, 0
Y	0, ½, 0
M	0, ½, ½
Z	0, 0, ½
Γ	0, 0, 0

Ab$_{50}$Or$_{50}$, cell 3

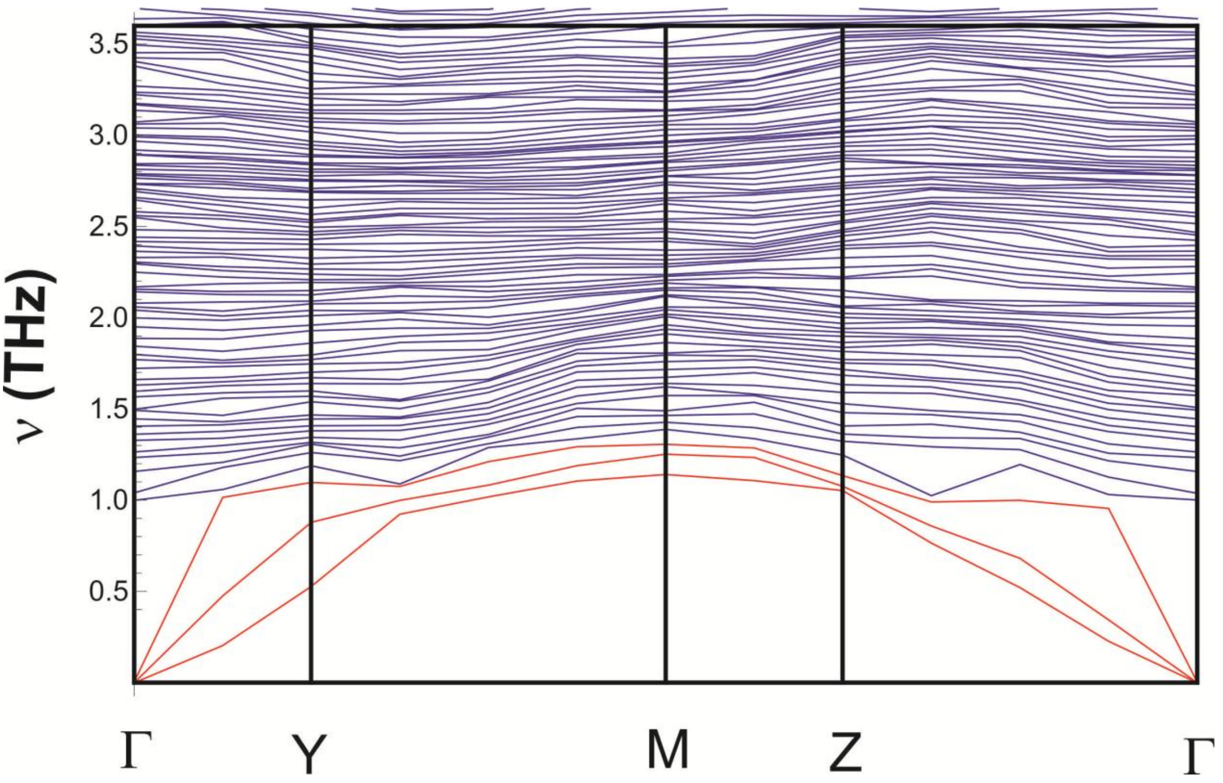

Ab$_{50}$Or$_{50}$, cell 4

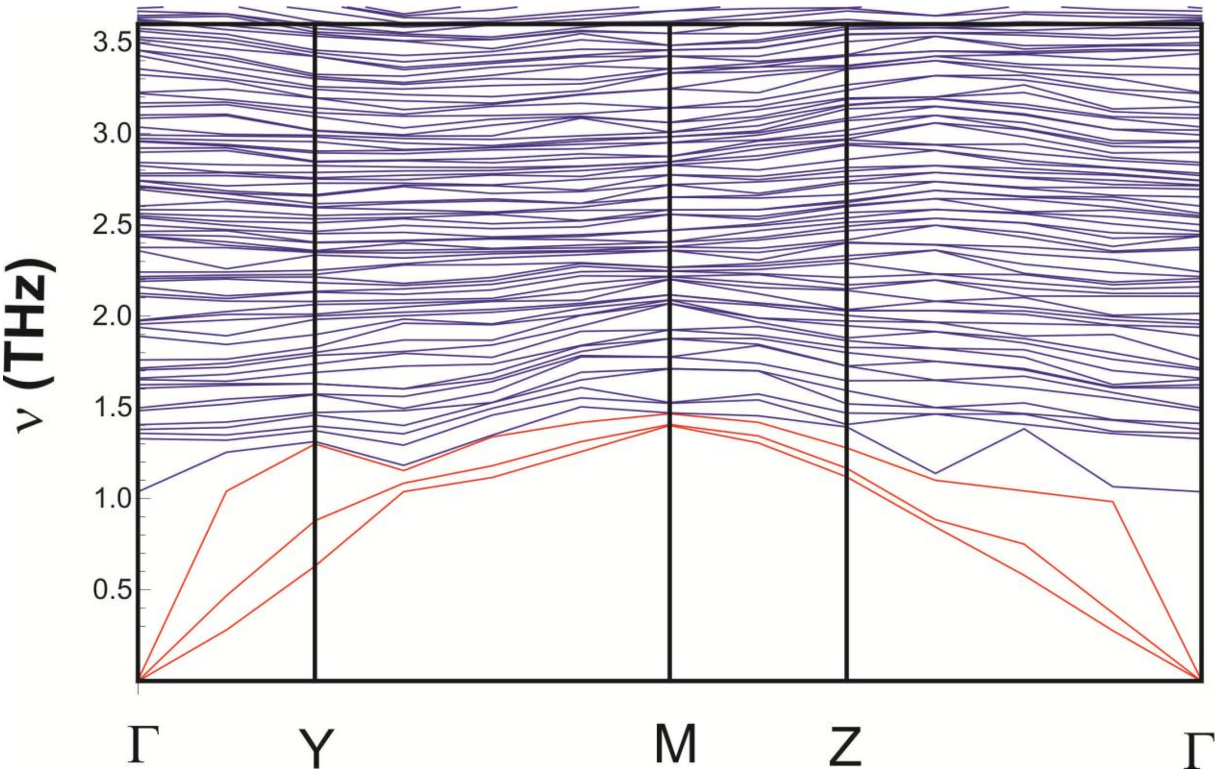

Supplement: Supplementary file 2 — Supplementary material 2 (PDF 1476 kb) [file 269_2014_715_MOESM2_ESM.pdf]
