# Supplementary material for: First-principles investigation of the lattice vibrations in the alkali feldspar solid solution
Source: Phys Chem Miner. 2014 Oct 11;42(3):243–9. doi: 10.1007/s00269-014-0715-8 (PMC4509561; doi:10.1007/s00269-014-0715-8)
Supplement: Supplementary file 3 — Supplementary material 3 (PDF 11 kb) [file 269_2014_715_MOESM3_ESM.pdf]

Appendix C:

Na – O and K – O bond lengths of investigated cells of the study "First-principles investigation of the lattice vibrations in the alkali feldspar solid solution" published by Artur Benisek, Edgar Dachs, Michael Grodzicki in Physics and Chemistry of Minerals.

Due to structural details of low albite and low microcline, Na was coordinated fivefold, whereas K sevenfold. The coordination numbers are in accordance with Downs et al. (1996). The Na – O and K – O bond lengths are listed in ascending order for low albite and low microcline, respectively.

Na – O bond lengths (Å):

	X_{Or}				
	0	0.25	0.5	0.75	1
Na 001 – O 002	2.30366	2.37390	2.46171	-	-
Na 001 – O 007	2.36410	2.40196	2.48539	-	-
Na 001 – O 019	2.39039	2.42894	2.50759	-	-
Na 001 – O 017	2.43864	2.47843	2.45919	-	-
Na 001 – O 001	2.52181	2.71383	3.05038	-	-
				-	-
Na 001 – O 008	2.96423	2.87844	2.71608	-	-
Na 001 – O 013	3.02264	2.77850	2.54396	-	-

	X_{Or}				
	0	0.25	0.5	0.75	1
Na 002 – O 010	2.30366	-	-	2.54604	-
Na 002 – O 015	2.36410	-	-	2.63809	-
Na 002 – O 027	2.39039	-	-	2.67897	-
Na 002 – O 025	2.43864	-	-	2.51907	-
Na 002 – O 009	2.52181	-	-	2.94529	-
Na 002 – O 016	2.96423	-	-	2.69003	-
Na 002 – O 005	3.02264	-	-	2.59607	-

K – O bond lengths (Å):

	X_{Or}				
	0	0.25	0.5	0.75	1
K 004 – O 026	-	-	2.53224	2.58011	2.62928
K 004 – O 009	-	-	2.68684	2.69174	2.74256
K 004 – O 025		-	2.69508	2.77873	2.77215
K 004 – O 031	-	-	2.69791	2.72794	2.84334
K 004 – O 032	-	-	2.92272	2.87152	2.86497
K 004 – O 011	-	-	2.72119	2.73597	2.86849
K 004 – O 021	-		3.17610	2.86655	2.88142
K 004 – O 012	-	-	3.05123	3.16208	3.01696
K 004 – O 022	-	-	3.07804	3.33197	3.33606

	X_{Or}				
	0	0.25	0.5	0.75	1
K 002 – O 010	-	2.46050	-	-	2.62928
K 002 – O 025	-	2.62422	-	-	2.74256
K 002 – O 009		2.64322	-	-	2.77215
K 002 – O 015	-	2.60702	-	-	2.84334
K 002 – O 016	-	2.91023	-	-	2.86497
K 002 – O 027	-	2.63152	-	-	2.86849
K 002 – O 005	-	3.12253	-	-	2.88142
K 002 – O 028	-	3.11886	-	-	3.01696
K 002 – O 006	-	3.10240	-	-	3.33606
